# Persistent differences between coastal and offshore kelp forest communities in a warming Gulf of Maine

**DOI:** 10.1371/journal.pone.0189388

**Published:** 2018-01-03

**Authors:** Jon D. Witman, Robert W. Lamb

**Affiliations:** Department of Ecology and Evolutionary Biology, Brown University, Providence, RI, United States of America; Sveriges lantbruksuniversitet, SWEDEN

## Abstract

Kelp forests provide important ecosystem services, yet coastal kelp communities are increasingly altered by anthropogenic impacts. Kelp forests in remote, offshore locations may provide an informative contrast due to reduced impacts from local stressors. We tested the hypothesis that shallow kelp assemblages (12–15 m depth) and associated fish and benthic communities in the coastal southwest Gulf of Maine (GOM) differed significantly from sites on Cashes Ledge, 145 km offshore by sampling five coastal and three offshore sites at 43.0 +/- 0.07° N latitude. Offshore sites on Cashes Ledge supported the greatest density (47.8 plants m^2^) and standing crop biomass (5.5 kg m^2^ fresh weight) of the foundation species *Saccharina latissima* kelp at this depth in the Western North Atlantic. Offshore densities of *S*. *latissima* were over 150 times greater than at coastal sites, with similar but lower magnitude trends for congeneric *S*. *digitata*. Despite these differences, *S*. *latissima* underwent a significant 36.2% decrease between 1987 and 2015 on Cashes Ledge, concurrent with a rapid warming of the GOM and invasion by the kelp-encrusting bryozoan *Membranipora membranacea*. In contrast to kelp, the invasive red alga *Dasysiphonia japonica* was significantly more abundant at coastal sites, suggesting light or dispersal limitation offshore. Spatial differences in fish abundance mirrored those of kelp, as the average biomass of all fish on Cashes Ledge was 305 times greater than at the coastal sites. Remote video censuses of cod (*Gadus morhua*), cunner (*Tautaogolabrus adspersus*), and pollock (*Pollachius virens*) corroborated these findings. Understory benthic communities also differed between regions, with greater abundance of sessile invertebrates offshore. Populations of kelp-consuming sea urchins *Stronglyocentrotus droebachiensis*, were virtually absent from Cashes Ledge while small urchins were abundant onshore, suggesting recruitment limitation offshore. Despite widespread warming of the GOM since 1987, extraordinary spatial differences in the abundance of primary producers (kelp), consumers (cod) and benthic communities between coastal and offshore sites have persisted. The shallow kelp forest communities offshore on Cashes Ledge represent an oasis of unusually high kelp and fish abundance in the region, and as such, comprise a persistent abundance hotspot that is functionally significant for sustained biological productivity of offshore regions of the Gulf of Maine.

## Introduction

Forests of Laminarian algae (kelp) provide many ecosystem services such as supporting nursery habitats, creating a productive, edible habitat for diverse assemblages of invertebrates and fish, contributing nutritive detritus for different groups of consumers, and dampening wave action [1, 2, 3]. Consequently, it is important to understand the processes determining spatio-temporal variation in kelp forests and the factors underlying their resilience. Local drivers of kelp abundance include sea urchin grazing [2, 4, 5, 6], storms [1, 7, 8], pollution [9], invasive species impacts [10, 11, 12] and indirect effects of top predators [13], while global drivers are linked to the El Niño Southern Oscillation [14,15] and ocean warming as a manifestation of climate change [3, 16]. By virtue of their relatively rapid rates of growth and recruitment, kelp are relatively resilient to disturbance [14, 17], although significant declines in kelp have occurred in concert with ocean warming in some areas off Western Europe and Australia [16, 18]. A global compilation of long-term data on kelp forest change indicated that while 38% of kelp forests are in decline globally, there is substantial regional variation in trends with kelp abundance increasing in 27% of regions [19]. This study pointed out that more research on patterns and drivers of kelp population change within regions is needed to resolve the potential large-scale erosion of ecosystem services provided by these important foundation species [19].

Coastal kelp forests in the Gulf of Maine and Atlantic Canada are typically zoned by depth with *Alaria esculenta* most abundant at shallow depths (<5 m) [20] and *Saccharina* species (*S*. *latissima*, *S*. *digitata*) dominating mid-depths (5–15 m) [7, 21–25]. Shotgun kelp *Agarum clathratum* thrives deeper in the rocky subtidal than *Saccharina* kelp [22, 25–28], and is particularly abundant in sea urchin dominated areas in the Gulf of St. Lawrence, Canada [29]. Kelp have declined since the 1970’s at several shallow coastal sites in the southwest coastal region of the Gulf of Maine [19, 21, 30].

**Fig 1:**
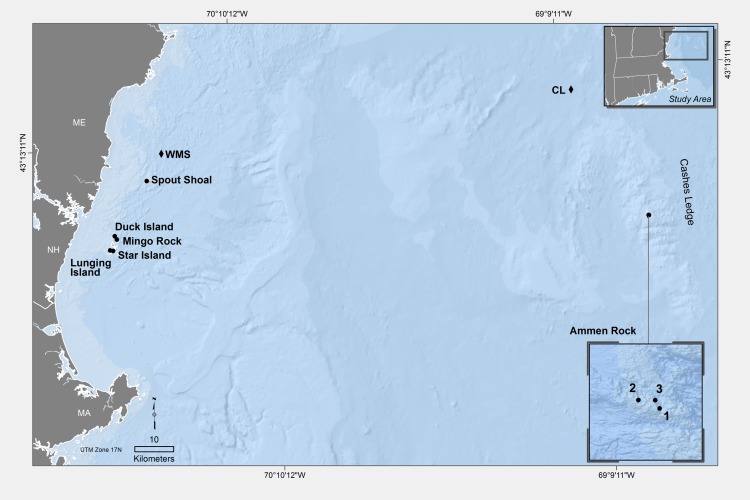
Map of the Gulf of Maine showing the location of the coastal and offshore (Cashes Ledge) sites. Cashes Ledge is located 145 km east of the New Hampshire coast with Ammen Rock at the center of the 29 km-long ridge. The site names are abbreviated as: AR1, AR2, AR3 designating Ammen Rock Sites 1–3 on Cashes Ledge and the coastal sites as: Spout Shoal (SS), Duck Island (DI), Mingo Rock (MR), Star Island (SI) and Lunging Island (LI). Sea Surface Temperature (SST) and wave height data were obtained from oceanographic buoy stations indicated by a diamond symbol with CL for the Cashes Ledge Buoy and WMS for the Western Maine Shelf Buoy. The scale bar is 10 km. Specific GPS coordinates for centrally located sites in the coastal and offshore region are: DI 43 00. 667 N x 70 60. 517 W, and AR1 42 53.4348 N x 068 56.6106 W. This map was produced by L. Carlson using Esri software licensed to Brown University.

Due to the logistic constraints of working offshore, less attention has been paid to the ecology of kelp forests in remote offshore regions in general [31], and specifically in the Western North Atlantic. In this study, we use the 100 m isobath [32] to define the boundary between coastal and offshore regions of the Gulf of Maine, as in Witman and Sebens [33]. The most prominent offshore kelp forest in the Gulf of Maine is located on Cashes Ledge, a 29 km long granitic ridge [34, 35] located 145 km east of coastal New Hampshire (Fig 1) [26, 27]. Deep (>30 m) kelp forests were initially described at Ammen Rock Pinnacle on Cashes Ledge as supporting the deepest laminarian kelp assemblages in the Western North Atlantic [26] with densities of *Saccharina latissima* and *Agarum clathratum* kelps reaching 7.0 and 1.0 individual plants per 1.0 m^2^ respectively at 30 m depth [27]. The occurrence of *Saccharina* kelp at greater depths (30 m) on Ammen Rock Pinnacle than in the coastal Gulf of Maine was attributed to greater light penetration in clearer offshore waters [26]. A larger, denser kelp forest was observed by J. Witman at shallower depths (<15 m) at Ammen Rock in 1977, but it was not studied. Ammen Rock refers to a uniquely shallow, broad platform several kilometers wide at the center of Cashes Ledge ridge, rising to within 10 m of the surface. Using multibeam backscatter techniques in a 8.8 km^2^ area on the western side of Ammen Rock, McGonigle et al. [36] estimated that the canopy volume of all benthic macrophytes (*Saccharina* sp., *Agarum* sp. combined) was 1.21 x 10^6^ m^3^ at < 24 m water depth. However, patterns of kelp forest community structure in this vast three-dimensional habitat have not been assessed by divers on Ammen Rock.

We hypothesized that the shallow kelp forest on Cashes Ledge supports unique, high biomass assemblages of fish as kelp habitats provide a complex three-dimensional habitat for demersal and mid-water rocky reef fishes [37]. In other areas, kelp forest fish distributions correlate with the availability of suitable habitat [38, 39], as well as with the abundance of associated benthic invertebrates and other food resources [40, 41]. As an offshore ridge in the midst of a gyre [42], Cashes Ledge is isolated, which may separate fish, kelp and benthic invertebrate populations living on Cashes Ledge from coastal areas. This isolation has contributed to the establishment of an endemic resident population of the unique variant red cod, *Gadus morhua*, [43] on Cashes Ledge. The Cashes Ledge area has been partially protected from some types of fishing (i.e. bottom trawling) as a Habitat of Special Concern by the United States Government since 2002 [44]. The combination of a complex, food-rich habitat created by kelp foundation species and moderate escape from fishing at Cashes Ledge may foster substantial populations of fish species that have suffered fisheries collapse elsewhere in the GOM region [44, 45].

Three main questions were addressed in this study: 1) Do offshore sites (Cashes Ledge) support populations and communities of macroalgae, fish and benthic invertebrates that differ in abundance, biomass and/or species composition from those at the same depths in the coastal Gulf of Maine? 2) Since warm water adversely affects kelp [16, 46], do these differences persist as the Gulf of Maine warms? and 3) Has the structure of the kelp, fish and benthic communities on Cashes Ledge changed since 1987? Our holistic approach involving kelp, fish and invertebrate and algal benthos is rarely taken in analyses of marine community structure, but it provides a unique opportunity to test for parallel trends across trophic groups and to identify species interactions that may influence the persistence of the kelp foundation species. In addition, our study informs trophic ecology and conservation by evaluating these sites as potentially persistent hotspots of kelp and fish abundance. While there is a growing list of pelagic and benthic hotspots, little is known about their persistence over time [47], which severely limits understanding their significance for food webs and for their contribution to other aspects of ecosystem functioning [48]. Here, we focus on spatial variation among the coastal and offshore sites and on decadal scale change on Cashes Ledge, because temporal trends in kelp abundance at shallow sites (< 10 m depth) in the southwest coastal region of the Gulf of Maine have already been documented [8,10, 19, 21, 30].

## Materials and methods

### Study sites

Data were collected by divers on research cruises to coastal sites during the summer months of 2014 and 2015, and on cruises to Cashes Ledge during summer 1987, 2012, 2014, 2015, and 2016 (Fig 1, Table 1). Due to rough seas and funding limitations, it was not possible to sample all coastal and offshore sites each summer. The 12–15 m depth range was chosen for study because it represents the shallowest depths of the kelp forest on Ammen Rock, enabling comparisons to coastal kelp forests at the same depth. Latitude was also held constant by selecting sites within the same latitudinal band. Consequently, the range of variation in latitude between the southern and northern extremes of the 5 coastal and 3 offshore sites was only 0.14 degrees of latitude, representing 20.5 km distance (Fig 1). The site names are abbreviated as: AR1, AR2, AR3 designating Ammen Rock Sites 1–3 on Cashes Ledge and the coastal sites as: Spout Shoal (SS), Duck Island (DI), Mingo Rock (MR), Star Island (SI) and Lunging Island (LI, Fig 1). In this paper, we use the terms “coastal” to refer to the coastal sites and “offshore” to refer to the Cashes Ledge region.

**Table 1:** Information on the sampling for kelp and fish. Number of replicate quadrats for urchin counts reported in S7 Table.

Region	Site	Sampling period	Kelp density (No. 1 m2 quadrats)	Kelp biomass (No. 1 m2 quadrats)	Fish biomass (No. 50 m2 transects)	Fish abundance (No. 10 minute video segments)
Offshore	Ammen Rock 1	July 1987	24	0	0	4
	Ammen Rock 1	August- September 2012	40	6	0	0
	Ammen Rock 1	June 2014	30	5	10	7
	Ammen Rock 1	June 2015	28	4	10	15
	Ammen Rock 2	June 2015	5	5	10	3
	Ammen Rock 2	June 2016	10	10	0	8
	Ammen Rock 3	June 2016	10	10	0	6
Coastal	Duck Island	June 2014	17	0	6	0
	Lunging Island	June 2014	10	9	0	0
	Lunging Island	June 2015	5	5	10	13
	Mingo Rock	June 2014	21	0	8	0
	Mingo Rock	June 2015	5	5	10	17
	Spout Shoal	June 2014	28	7	3	0
	Spout Shoal	June 2015	5	5	10	4
	Star Island	June 2014	24	4	7	8
	Star Island	June 2015	5	5	10	4

### Environmental variables

Variation in sea surface temperatures and wave heights was analyzed to characterize the physical environment of the GOM sites during the study period and its potential to affect the kelp forest community. At the scale of the entire GOM, SST anomalies were calculated from 1980–2016 by downloading temperature data from the Met Office Hadley Centre (HadISST www.metoffice.gov.uk/hadobs/hadisst/)). A trend line was fit across the time series using a rolling function based on the median anomaly using a 24-month window with the R program *stat_rollapplyr()*. Comparisons of coastal and offshore SST trends during 2002–2017 were made by downloading data from oceanographic buoy stations (Western Maine Shelf http://www.ndbc.noaa.gov/station_page.php?station=44030) near the coastal Spout Shoal site and from a location near the northern edge of Cashes Ledge (http://www.ndbc.noaa.gov/station_page.php?station=44005, Fig 1). Temperatures were measured at 1.0 m depth at both buoys. Wave height data were also obtained from both buoy stations during the sampling years (1987, 2012, 2014–2016). No wave data were available for the Western Maine Shelf buoy in 1987.

### Quantitative surveys

#### Kelp

Quadrat sampling was conducted to estimate the abundance of kelp at 12–15 m depth at the study sites (Table 1). The procedure consisted of randomly tossing three-sided 1.0 m^2^ quadrats onto the rock substrate as in Witman [7]. All kelp plants longer than 10.0 cm in the quadrat were either counted *in situ* or scraped off the substrate with a putty knife and placed in a mesh bag. Biomass was measured directly by weighing all individual kelp in several samples. These measurements were used to establish regression equations of total length against weight determined for each species from samples collected underwater (where y = fresh weight biomass in grams and x = length in cm: *S*. *latissima* y = 0.00421 * x^1.951^, r^2^ = 0.93, n = 198; *S*. *digitata* y = 0.000942 * x^2.4541^, r^2^ = 0.76, n = 150; *A*. *clathratum* y = 0.013511 * x^1.9486^, r^2^ = 0.85, n = 103). The resulting equations were then used to calculate biomass for individuals for which only lengths were available. Kelp plants were blotted to remove excess water prior to weighing. As such, biomass values represent fresh weight as in Gevaert et al. [49]. The three dominant species of kelp in the Gulf of Maine, *Saccharina latissima*, *Saccharina digitata*, *and Agarum clathratum* were sampled by these procedures. The fucoid alga *Desmarestia aculeata* was present in low abundance at most sites but the density and biomass of this species was not determined since it was difficult to collect whole plants intact. To increase the spatial extent of kelp assessment at Cashes Ledge beyond quadrat sampling (Table 1), the percent cover of kelp species was analyzed from 30–50 m long video transects taken across the canopy in 2014 and 2015. The analytical procedure consisted of using random numbers to select individual still frame images corresponding to a time on the video. A screen of 200 random dots was then superimposed on the still image using Adobe Photoshop ™ software. The proportion of dots falling on *S*. *lattisima*, *S*.*digitata*, and *D*. *aculeata* was determined, yielding an estimate of percent cover.

Two methods were used to assess the percent cover of the invasive bryozoan, *Membranipora membranacea* on kelp at the Cashes Ledge sites. The first was based on a video taken in 1987 and on video transects from 2014 and 2015, using the same random dot method as above, but for *Membranipora* cover, on randomly selected still images. The second method involved randomly selecting digital photographs of the kelp forest taken in August—September 2012 by B. Skerry. The percent cover of the conspicuously white *M*. *membranacea* colonies on the kelp plants was estimated by the same random dot technique (as above) applied to the images selected by either method. There was striking variation in the cover of *Membranipora* on the kelp between sampling years, however, the use of two different methods precluded statistical comparisons.

The kelp forest at the AR1 site was also sampled in July 1987 by the same quadrat methodology (Table 1). Combining the 1987 data with the data on kelp density from AR 1 during 2012, 2014, and 2015 enabled us to analyze long-term changes in kelp density on Cashes Ledge.

#### Benthic community structure

The diversity and abundance of epifaunal invertebrates and understory macroalgae were assessed by photographing 0.25 m^2^ quadrats of the rocky bottom (n = 24–43 quadrats per site) at randomly determined locations with a quadrapod [5]. Sampling was conducted at AR1 in July 1987 and at coastal (SS, MR, ST, LI) and Cashes Ledge (AR1, AR2) sites in 2015. The resulting photo quadrats were projected on a computer screen and a layer of 200 random dots was then superimposed on the image using Adobe Photoshop ™ software. The proportion of dots falling on different species of invertebrates and algae was determined, yielding an estimate of percent cover.

Because of their ability to radically alter the local distribution of kelp, the abundance of sea urchins, *Stronglyocentrotus droebachiensis*, was censused in the same 1.0 m^2^ quadrats surveyed for kelp abundance and in additional, randomly placed quadrats during 1987, 2014 and 2015. A random collection of 57 urchins was made from the quadrats at Star Island in 2014 to estimate their body size. During the quadrat sampling, it was noted whether the urchins were smaller or larger than 3.0 cm test diameter.

#### Fish

Fish abundance was measured using two complementary techniques: underwater visual census, which provides a snapshot of a large area and facilitates estimates of fish biomass, and stationary video, which provides a longer temporal window of a single area and removes potential effects of diver presence on fish behavior. Visual censuses were performed along 30–50 m long horizontal transects stretched across the bottom with the observing diver swimming within the same 12–15 m depth zone (Table 1). Transect starting points and directions were selected haphazardly. Fish were counted along each transect in 10.0 x 5.0 m blocks in order to compare different total areas surveyed. Within these blocks, all fish were counted, identified to species, and a visual estimate was made of their size. All size estimates were made by a single researcher experienced in underwater fish census techniques (R. Lamb) [50]. Fish total lengths were converted to biomass using established length-weight ratios using the equation W = a x L^b^, where W is weight in grams, L is fish length in cm, and a and b are constants specific to each fish species describing its exponential mass increase with length [51–53].

The second technique involved placing stationary video cameras (GoPro® Hero4) at each site for 1 hour in between dives. The cameras were mounted on tripods 80 cm above the substrate and haphazardly placed to look down onto the kelp bed and over the reef. A triangular 30.0 m^2^ viewing area was established by extending a transect tape 6 m deep and 10 m across at the most distant point from the camera. All fish passing through the area were observed in time blocks of 10 min each. We recorded eight blocks per site and date, with cameras separated by at least 20.0 m distance and with blocks separated by at least 5 minutes of interval time. For each species observed we recorded MaxN, the maximum number of individuals observed in a single frame of the video. This is an established method that provides the most conservative possible abundance estimate since it prevents counting the same individual twice [54]. Dates and locations of fish censuses are reported in Table 1. Fish abundance was measured at the AR 1 site on Cashes Ledge in July 1987 by a single deployment of a stationary video camera so only qualitative comparisons were made to the 1987 data.

### Statistical analyses

We tested for differences in kelp abundance (biomass, density) between the study sites by 1-way ANOVA on log x +1.1 transformed data. Tests were repeated for each sampling year rather than in a two-factor design (site x year), since different sites were sampled during different years. Temporal variation in kelp density at the AR1 site was also analyzed by 1-way ANOVA (log x +1.1 transformation) using the factor year (1987, 2012, 2014, 2015). Post-hoc Tukey’s Honest Significant Difference tests (HSD) revealed which sites differed after the main effect was evaluated. The effect of year (2014, 2015), site and their interaction on urchin densities was analyzed by two-way ANOVA using data from the 4 sites that were sampled during both years (AR1, SI, SS, MR). Repeated measures ANOVA was unnecessary as the quadrats were sampled in different places on the bottom each year.

After observing that *Saccharina digitata* rarely occurred in dense stands of *Saccharina latissima* on Cashes Ledge, we tested a corollary of the hypothesis that *S*. *digitata* density is limited by interspecific competition with *S*. *latissima*, a known competitive dominant [55], on the scale of 1.0 m^2^ areas by non-linear regression analysis. The analysis was made using quadrat counts of both species pooled from the AR sites in 2015 and 2016.

Data on fish biomass (transects; g per 50 m^2^) and abundance (videos; MaxN per 10 min) were analyzed by two-way ANOVA to test for the effects of site and year on log (x+1)-transformed data for each species. Pairwise comparisons were then performed for significant factors and interaction terms using Tukey’s HSD. All analyses were performed in R [56] or in JMP^R^ Pro 12.1 statistical software.

We used the *vegan* package in R to examine the multivariate composition of rocky subtidal communities consisting of benthic macroalgae and invertebrates at each coastal and offshore site using non-metric Multi-Dimensional Scaling (nMDS). Prior to analysis, the data were arcsine square-root transformed. Species and site ordination values were created using the Bray-Curtis dissimilarity index. The model converged after 19 iterations, with an acceptable stress value of 0.191. Differences in the multivariate structure of benthic communities between sites were revealed by Permutational Analysis of Variance (PERMANOVA) using the function *adonis()*. Similarity percentage analysis (SIMPER) was performed to examine the primary species contributing to variation between the onshore and offshore sites.

Cashes Ledge encompasses the only shallow (< 15 m depth) rocky subtidal habitat in the entire offshore region of the Gulf of Maine, which is Ammen Rock. The Ammen Rock area is large, measuring considerably more than 8.8 km ^2^ in area [36], enabling benthic communities to be sampled at three independent sites (AR1-AR3, Fig 1). We did not formally test for the effects of region (i.e., onshore vs offshore) by parametric statistics because there was only a single offshore and a single coastal region. Rather, site was used as the main factor in parametric statistical analyses. The effects of region on benthic community structure was, however, tested by the non-parametric PERMANOVA, as indicated above.

## Results

### Environmental conditions

**Fig 2:**
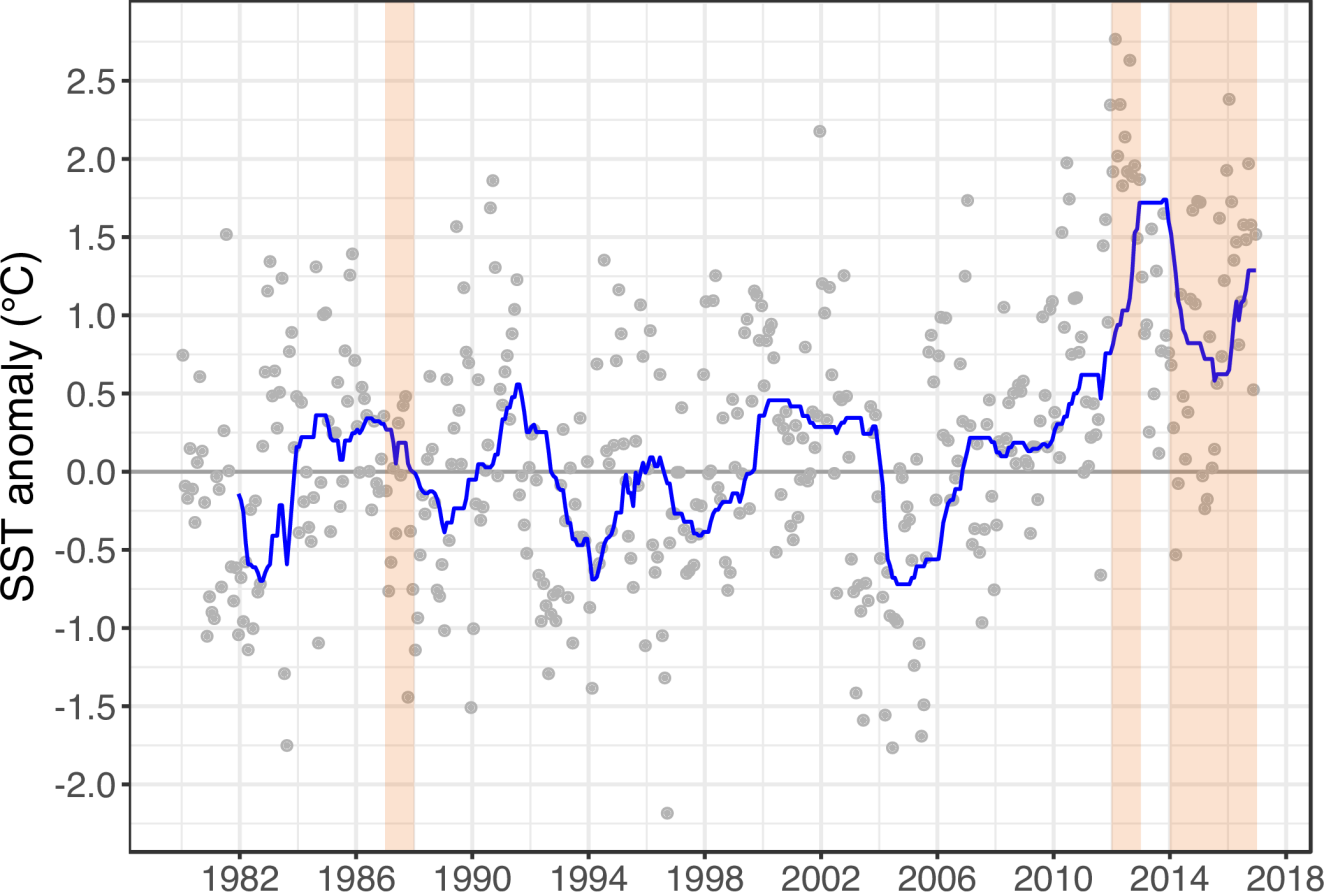
Sea surface temperature (SST) anomalies for the Gulf of Maine between 1980 and 2016. Data were compiled from the Met Office Hadley Centre data set (HadISST). Orange shaded bars correspond to the years that kelp forest communities were sampled on Cashes Ledge. Note that SST anomalies were consistently positive from late 2006 to 2016, and that a record 2.75° C warm anomaly occurred in 2012. A trend line (blue) was fit across the time series using a rolling function based on the median anomaly using a 24-month window with the R program *stat_rollapplyr()*. Grey dots represent individual SST anomaly data points.

Temperature analysis indicated that the Gulf of Maine has warmed since the study began in 1987 (Fig 2). Positive SST anomalies have prevailed since 2006, with a record anomaly in summer 2012 of 2.75° C above the long term (1980–2000) median, and a 2.25° C positive anomaly in 2016. Regional differences in SST were also apparent, as temperatures were generally warmer on Cashes Ledge than in the coastal zone (S1 Fig). Minimum temperatures dropped below 2.5° C only once (winter 2004) on Cashes Ledge, whereas this occurred in 4 years at the coastal Western Gulf of Maine buoy (2003–2005, 2015, S1 Fig). The opposite pattern occurred for maximum temperatures, which exceeded 22.5° C during 4 years at Cashes Ledge (2010, 2012, 2015, 2016) but during only 1 year in the coastal zone (2013). Temperatures peaked during July—August 2012 with a record 24.5° C on Cashes Ledge (S2 Fig). For short periods of time, SST was as much as 8.0° C higher on Cashes Ledge than in the coastal zone in 2012 (S2 Fig). The 99^th^ percentile maximum temperatures were 0.98 to 2.77° C higher on Cashes Ledge than in the coastal zone (Western Maine Shelf, S1 Table).

The oceanographic buoy data indicated that the Cashes Ledge area experiences higher waves than the coastal Western Maine Shelf buoy near Spout Shoal. This is apparent from the consistently lower cumulative wave distributions from the coastal as compared to the Cashes Ledge buoy (S3 Fig). The 95^th^ and 99^th^ cumulative percentiles of wave heights were higher on Cashes Ledge, by as much as 0.91 m within a sampling year, than in the coastal region (S2 Table). Extreme wave heights peaked at 5.50 m on Cashes Ledge in 1987. The comparatively greater wave heights on Cashes Ledge are accentuated by the fact that the water depth below the Cashes buoy is 3 times deeper than at the coastal Western Maine Shelf buoy (S2 Table), indicating that wave heights will be more amplified by shoaling with the bottom [57] at the Western Maine Shelf than at the Cashes buoy.

### Kelp

**Fig 3:**
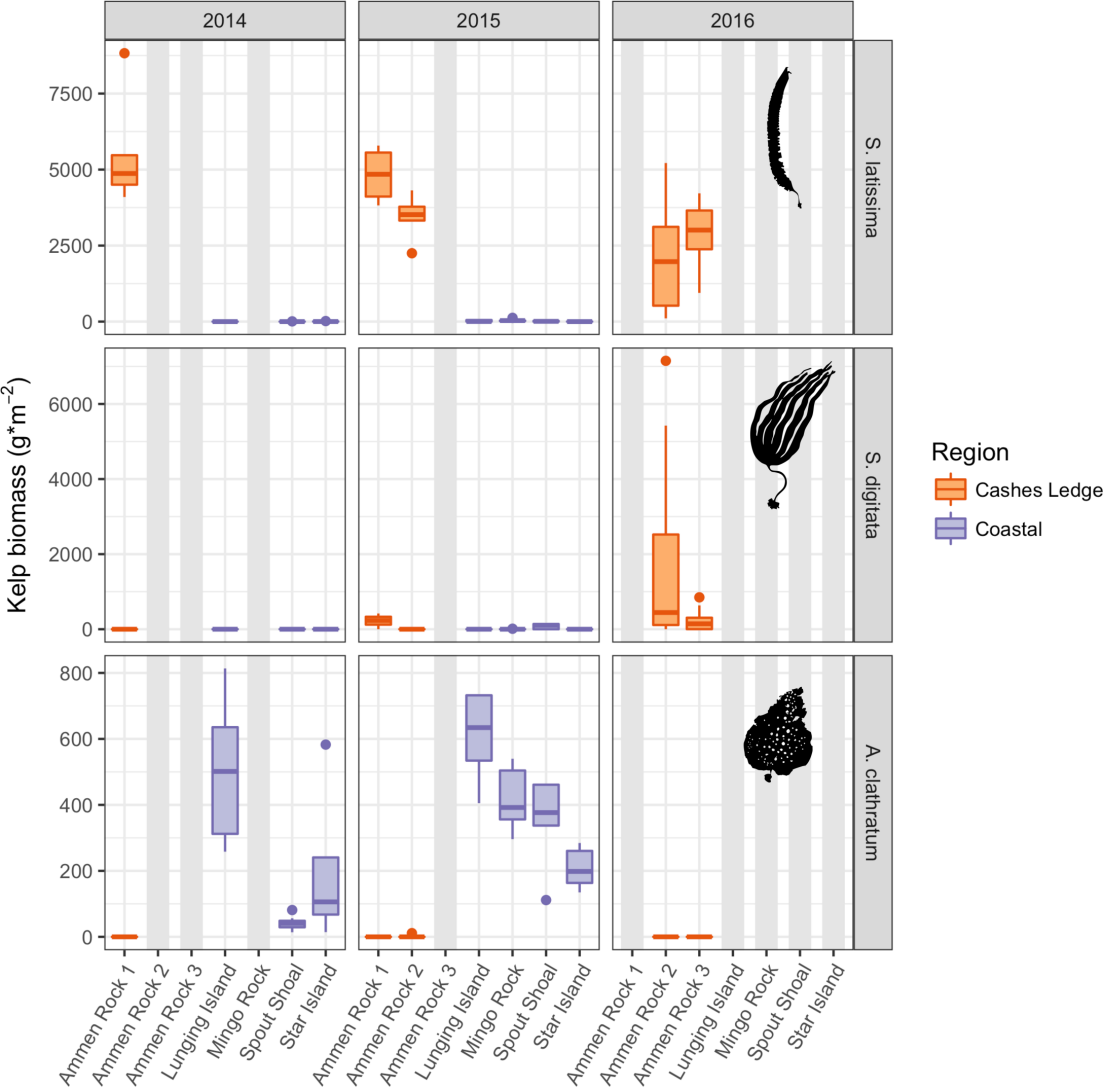
Kelp biomass. Boxplots display the median value of the data as a horizontal bar, the first and third quartiles of the data (25th and 75th percentiles) as a shaded box around the mean, 1.5x the interquartile range (IQR) as a vertical line, and all outliers beyond +/- 1.5 IQR as individual dots. Ammen Rock sites on Cashes Ledge are colored orange while data from coastal sites are purple. These graphical representations are repeated in all subsequent plots. Note that the y-axis ranges are different for each species. Grey shaded areas indicate sites that were not sampled in that year.

The average biomass of *Saccharina latissima* varied by 3 orders of magnitude between the coast and offshore, from an average of 1.4 to 5,553.8 g per 1.0 m ^2^ at SS and at AR1, respectively (Fig 3). During both years that offshore and coastal sites were sampled, 1-way ANOVA indicated highly significant differences in *S*. *latissima* biomass among sites (p < 0.0001, 2014, 2015, S3 Table). The pattern of significantly higher kelp biomass on Cashes Ledge than at coastal sites persisted when replication was increased in 2015 by sampling another site on Cashes Ledge (AR2) and another coastal site (MR, S3 Table). Tukey post hoc tests indicated that *S*. *latissima* biomass was significantly higher at AR1 than at the 3 coastal sites during both 2014 and 2015, and that in 2015 the biomass of this species at AR2 was also significantly higher than at all coastal sites (S3 Table). There was no difference in *S*. *latissima* biomass between the two sites sampled on Ammen Rock in 2015 and 2016. Overall, there was no significant difference in biomass of *S*. *latissima* among the coastal sites in 2014 and 2015 (S3 Table). High *S*. *latissima* biomass at the Ammen Rock sites persisted in 2016 (Fig 3).

Spatial patterns of congeneric *Saccharina digitata* biomass were broadly similar to that of *S*. *latissima*, as *S*. *digitata* biomass was, in most comparisons, significantly higher offshore at the Ammen Rock sites than at the coastal sites in 2015 (Fig 3, S3 Table). There was however, greater temporal and spatial variability in *S*. *digitata* than in *S*. *latissima* biomass. There was an overall trend of increasing *S*. *digitata* biomass at AR1 from 2012 to 2015 with high biomass in 2016 (AR2, AR3, Fig 3) as well. Low biomass *S*. *digitata* stands occurred at two coastal sites, SS and MR. The biomass of *S*. *digitata* at MR was not significantly different from AR2, and similarly, the biomass at SS was not significantly different from that at AR1 and AR2 (S3 Table).

Patterns of the shotgun kelp, *Agarum clathratum*, biomass were the opposite of *S*. *latissima*, as *A*. *clathratum* biomass was significantly higher at the coastal sites than at all of the offshore sites on Ammen Rock during 2014 and 2015 (Fig 3, S3 Table). *A*. *clathratum* dominated kelp assemblages in the coastal zone at the 12–15 m depths sampled. Like *S*. *digitata*, *A*. *clathratum* biomass increased from 2012 to 2015. There was significant variation in *A*. *clathratum* biomass between coastal sites in 2014, with greater biomass at SS than at LI (S3 Table).

**Fig 4:**
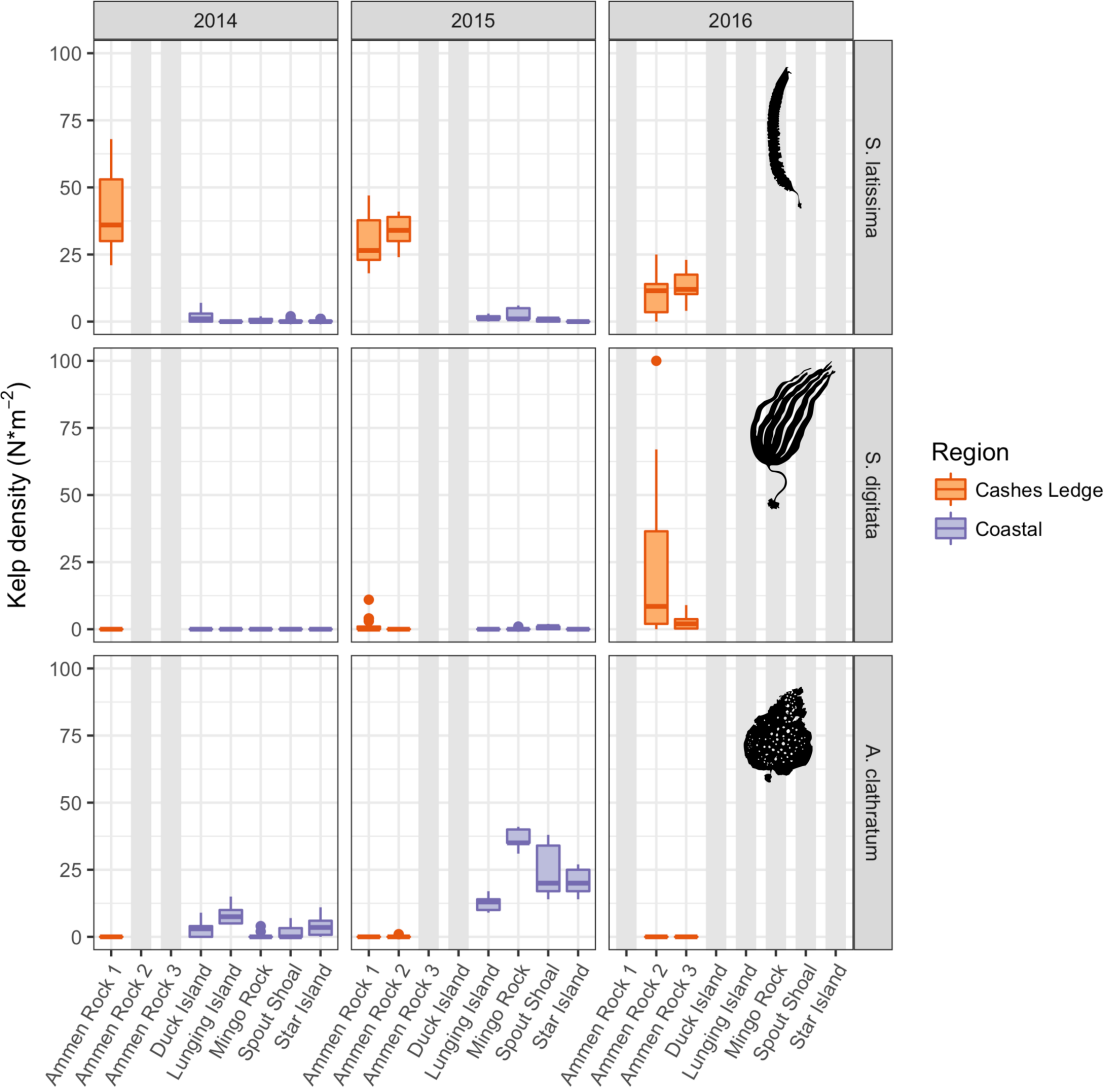
Kelp density. Data shown are number of individual kelp stipes per 1.0 m^2^. Boxplots as in Fig 3.

*Saccharina latissima* consistently attained the densest stands of any kelp species, reaching maximum average densities of 47.8 plants per 1.0 m^2^ at AR1 in July 1987. During 2014 and 2015, *S*. *latissima* was significantly more abundant at the Ammen Rock sites on Cashes Ledge than at each of the coastal sites (Fig 4, S4 Table). During the years that both coastal and offshore sites were sampled, average *S*. *latissima* density was up to 162 times higher at Ammen Rock (AR1 2014) than at any of the coastal sites where this species was present (SI 2014). The Duck Island site stood out among coastal sites as it supported significantly higher densities of *S*. *latissima* (1.7 plants per 1.0 m^2^) than all four other coastal sites (Fig 4, S4 Table).

**Fig 5:**
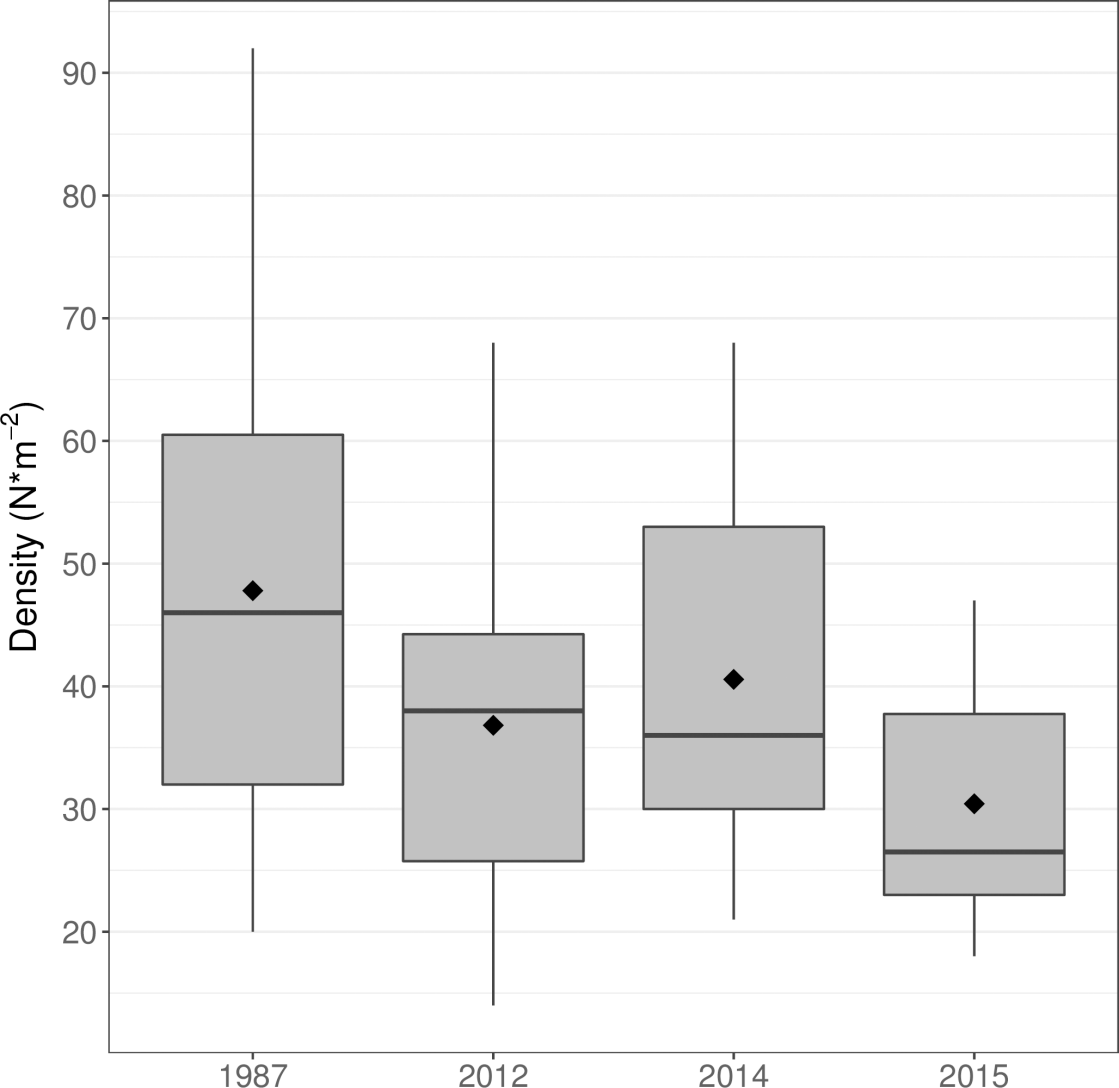
Temporal variation in average *S*. *latissima* density at Ammen Rock Site 1 during four sampling periods from 1987 to 2015. A significant 36.2% reduction of average kelp density occurred between 1987 and 2015 (S4 Table). The low kelp densities in 2012 occurred during anomalously warm sea surface temperatures (Fig 2, S1 and S2 Figs) when the kelp plants were extensively covered by the invasive bryozoan *Membranipora membranacea* (Fig 6E, S5 Table). The boxplots display the median value of the data as a horizontal bar, the first and third quartiles of the data (25th and 75th percentiles) as a shaded box around the mean, 1.5x the interquartile range (IQR) as a vertical line, and all outliers beyond +/- 1.5 IQR as individual dots. The diamond symbol represents the mean value.

A highly significant, 36.2% decrease in *S*. *latissima* density occurred at AR1 between 1987 and 2015 (Fig 5, 1 way ANOVA, p = 0.0007; followed by Tukey’s test, S4 Table). Kelp densities also decreased significantly between 2014 and 2015 (p = 0.033, S4 Table) and the trend of declining *S*. *latissima* density between 1987 and 2012 was nearly significant (p = 0.054, S4 Table).

**Fig 6:**
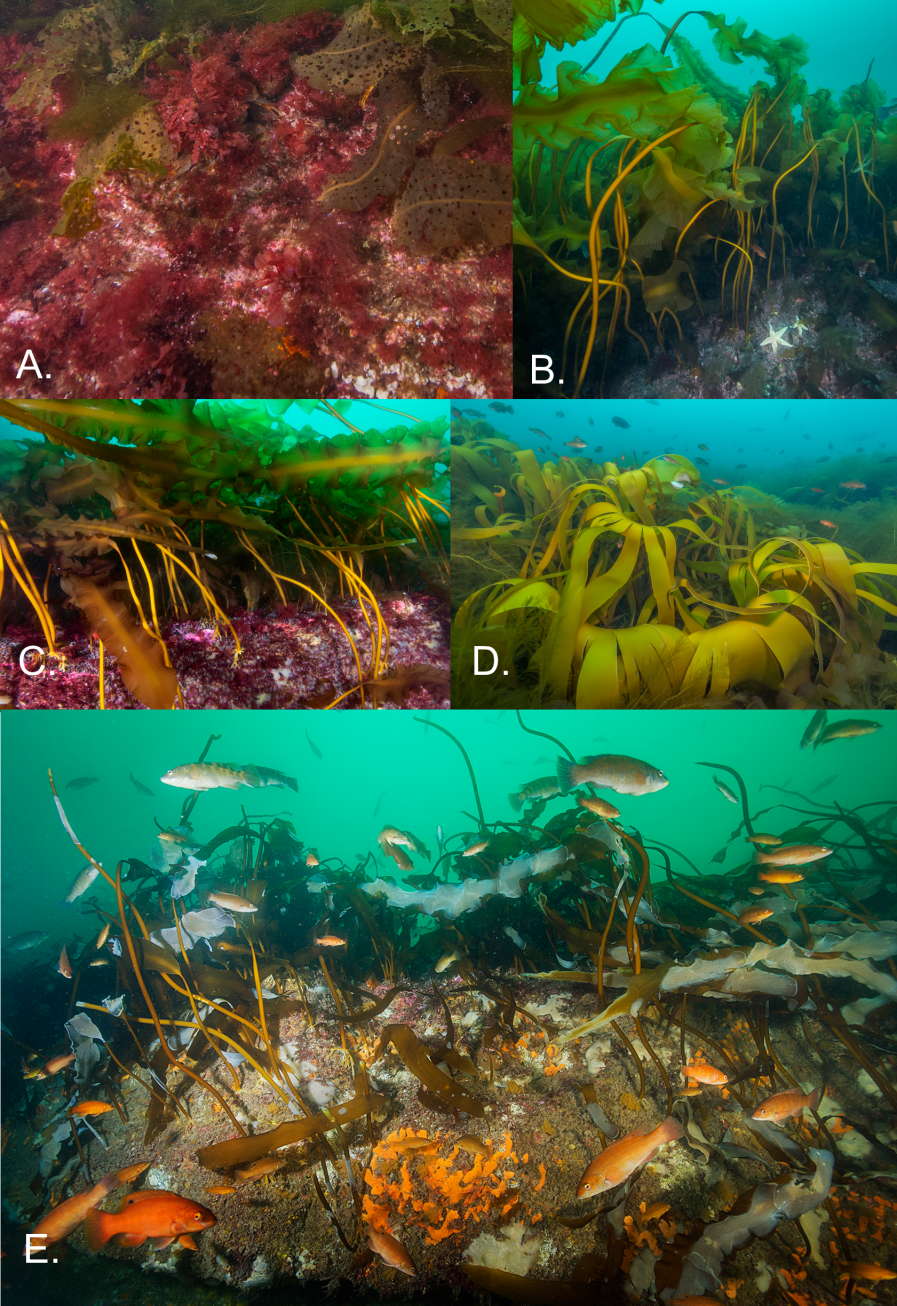
Kelp communities in the coastal zone and offshore on Cashes Ledge. **A**. view of common macroalgae at a coastal site, Mingo Rock. Blades of the dominant shotgun kelp *Agarum clathratum* are visible in addition to the red algae *Euthora* sp. and the invasive *Dasysiphonia japonica*. **B.** Side view of *Saccharina latisssima* forest at AR site 2 on Cashes Ledge, showing the tall stature and complexity of the habitat created by this foundation species. **C.** View of *S*. *latissima* forest at AR site 1 showing understory red algae and small colonies of the ascidian *Aplidium constellatum*. **D**. Patch of *Saccharina digitata* kelp at AR site 2 in 2015. *S*. *digitata* density is inversely related to that of *S*. *latissima*, suggesting interspecific competition. Small fish in the background are cunner (*Tautogolabrus adspersus*). **E**. Photo of a deteriorating assemblage of *S*. *latissima* kelp at AR1 site during the anomalously warm summer of 2012. Note the high incidence of kelp covered with white colonies of the invasive bryozoan *Membranipora membranacea*, kelp stipes lacking blades and abundant copper colored fish (cunner). Photo credits: Fig 6A, B and D by Brett Seymour, Fig 6C and E by Brian Skerry.

Over this same time period, the invasive bryozoan *M*. *membranacea* colonized the Cashes Ledge kelp forest, covering 28% of *S*. *latissima* kelp plants during the anomalously warm summer of 2012 (Fig 6E), then declining to less than 1.0 to 6.0% cover at two Cashes Ledge sites in 2014–2015 (S5 Table).

Small patches of *Saccharina digitata* were observed at 12–15 m depths at AR1 on Cashes Ledge in 2014 and 2015 (Fig 6D). By 2016 it was abundant at 2 sites on Ammen Rock (AR2, AR3) in densities exceeding that of *S*. *latissima* at AR2 (Fig 4). One-way ANOVA on the factor year followed by Tukey tests indicated that *S*. *digitata* densities increased significantly from 1987 to 2015 at the AR1 site (F = 9.27, 3, 118 df, p < 0.0001, S4 Table). Both quadrat sampling and video transects revealed an increase in *S*. *digitata* abundance between 2014 and 2016 (S4 Table, Table 2, Fig 4). The percent cover of the fucoid alga *Desmarestia aculeata* increased to 17% between 2014 and 2015 (Table 2).

**Table 2:** Percent cover of macroalgae at Cashes Ledge sites (AR1, AR2) in 2014 and 2015. Data are means and standard deviations (in parentheses) of the kelp canopy assessed by “freeze frame” images sampled at random locations along 30 m long video transects at 12–15 m depth. *D*. *aculeata* is the fucoid alga *Desmarestia aculeata*. Each image sampled measured 2.6 to 3.0 m^2^ in 2014, and 3.0 to 3.4 m^2^ in 2015.

Site	Year	*S*. *latissima*	*S*. *digitata*	*D*. *aculeata*	Sample size (n)
AR 1 (transect 1)	2014	99.5 (1.3)	0.2 (1.1)	0.1 (0.6)	22
AR 1 (transect 2)	2014	100 (0)	0	0	25
AR 1	2015	72.5 (27.9)	9.6 (16.7)	17.6 (26.7)	29
AR 2	2015	78.0 (23.0)	4.0 (10.1)	17.8 (22.8)	23

Patterns of *Agarum clathratum* density and biomass were the opposite of *S*.*latissima* (Figs 3 and 4) with one exception (MR, S4 Table). *A*. *clathratum* was more abundant at all coastal sites than at offshore sites in 2014 and this pattern held without exception in 2015. Among the coastal sites, Tukey tests indicated that significant differences in *A*. *clathratum* densities occurred between the site with the lowest densities (LI) and 3 other coastal sites including MR, which contained the densest *A*. *clathratum* stands (36.4 plants per 1.0 m^2^, Fig 4, S4 Table).

### Interspecific patterns

The density of *Saccharina digitata* was significantly and inversely related to that of *Saccharina latissima* at the Ammen Rock sites by the equation *S*. *digitata* density (y) per 1.0 m^2^ = 56.987–38.981 * log [*S*. *latissima* density (x) per 1.0 m^2^ + 1.1] with an r^2^ of 0.764 (n = 53).

There was a nearly 20-fold difference in the average percent cover of the invasive red alga *Dasysiphonia japonica* [58] across the study sites in 2015 ranging from 4.2% at AR1 on Cashes Ledge to 81.9% at a coastal site, Spout Shoal (Table 3). One-way ANOVA using the factor site followed by Tukey tests indicated that *Dasysiphonia* cover varied significantly by site (p < 0.001, S6 Table). This invasive red alga was significantly more abundant at all onshore sites than at either of the Ammen Rock sites (S6 Table). *Dasysiphonia* was not present on Cashes Ledge (AR1) in 1987.

**Table 3:** Percent cover of the invasive red alga *Dasysiphonia japonica* at the study sites. Data represent the mean +/- one Standard Error (SE) percent cover. Sample sizes are numbers of 0.25 m^2^ area photo quadrats analyzed. Data are from 2015 unless noted otherwise.

Study Site	Mean Percent Cover	Standard Error		Sample Size (n)
Lunging Is.	20.2	2.3		31
Star Is.	24.0	1.9		24
Mingo Rock	23.4	2.4		38
Spout Shoal	81.9	3.1		24
Ammen Rock 1	4.2	1.1		43
Ammen Rock 2	11.9	1.8		25
Ammen Rock 1 1987	0	0		24

### Sea urchin abundance

Patterns of sea urchin (*S*. *droebachiensis*) densities were the inverse of *S*. *latissima* kelp as they were consistently more abundant at the coastal sites than offshore on Cashes Ledge. For example, only 1 small urchin (0.75 cm TD) was found at any of the Ammen Rock sites (AR 1 2015) in a total of 146 quadrats (1.0 m^2^) sampled during 1987, 2012, 2014, 2015 and 2016, while urchin densities peaked at a mean of 36.2 individuals per 1.0 m^2^ at the coastal SI site in 2015 (S7 Table). Significant site variation in urchin densities occurred in 2014 (1-way ANOVA, p < .0001, F 7.99, 4, 50 df, data log x + 1.1 transformed). Post hoc Tukey HSD tests indicated that urchin densities were significantly higher at SI (Q = 2.83, alpha 0.05) than at all other sites at this time. Similarly, significant site variation in urchin densities also occurred in 2015 (1-way ANOVA (p < .0001, F = 202.7, 5,73 df) with Tukey HSD tests indicating that urchin densities at SI were significantly higher (Q = 2.93, alpha 0.05) than at all other sites (LI, SS, MR, SS, AR1, AR2). Urchin densities on Cashes Ledge were significantly lower than at all coastal sites. A factorial ANOVA testing the effects of Year, Site and their interaction on urchin densities at the subset of 4 sites sampled during both 2014 and 2015 (SI, MR, SS, AR1) indicated the same site effects as above but also revealed significant effects of Year (p < .0001, F = 33.3), and a Year by Site interaction (p < .0001, F = 41.9). Post hoc Tukey tests demonstrated that the significant effect of year (higher in 2015) was caused by exceptionally high urchin recruit densities at SI in 2015 (t = 1.98, p < .05).

The size structure of urchin populations was dominated by small individuals, as 66 out of 67 (98.5%) urchins at the coastal sites were smaller than 3.0 cm test diameter (TD) in 2014 and the average TD of urchins at the SI site then was 1.1 cm (0.7 SD, n = 57). In 2015, all of the 382 urchins counted were smaller than 3.0 cm TD.

Using Balch and Scheibling’s [59] finding that a 1 year old sea urchin is less than 0.8 cm TD, and that juvenile urchins are less than 1.6 cm TD, we estimate that the single urchin found at AR 1 in 2015 (0.75 cm TD) recruited in the previous year and that the majority of urchins in the coastal zone were juveniles or approximately 2 years old.

### Multivariate structure of benthic communities

**Fig 7:**
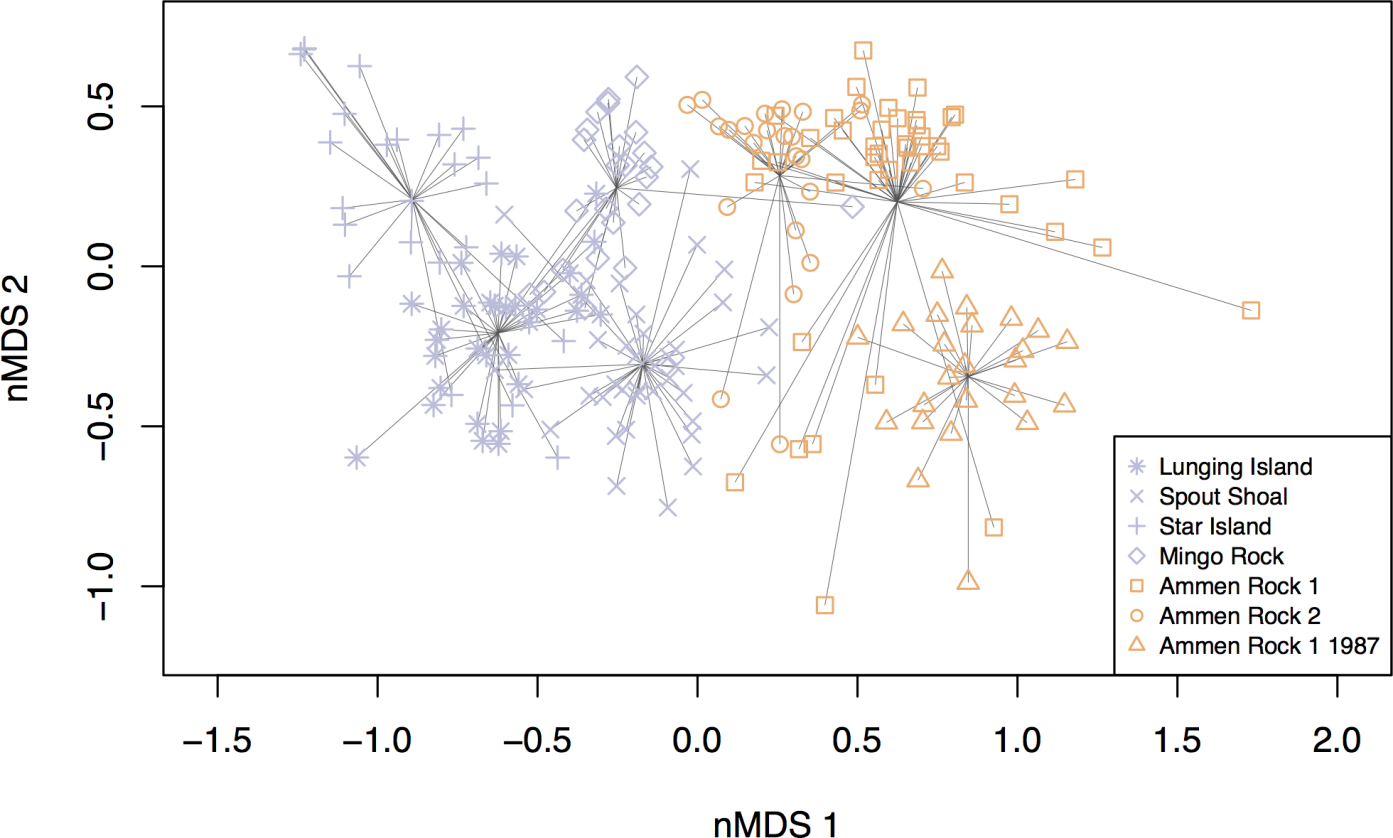
nMDS plot indicating differences in benthic community structure (sessile invertebrates, understory algae) between coastal and offshore sites on Cashes Ledge (Ammen Rock 1, 2, Ammen Rock 1 1987). Note the spatial segregation between coastal sites (colored purple) and offshore sites (colored orange), and the shift in the composition of the benthic community at the Ammen Rock sites between 1987 and 2015, when Ammen Rock 1 and 2 sites were sampled. Each individual data point represents the benthic community sampled in a 0.25 m^2^ quadrat. Replication consisted of the following number of photo quadrats per site: LI 31, SS 24, SI 24, MR 38, AR1 43, AR2 24 and AR1 1987 24.

Non-metric multidimensional analysis followed by PERMANOVA revealed significant differences in clusters of species composition among sites (Fig 7, r^2^ = 0.59; p <0.001, S8 Table), resulting in a clear separation between coastal and offshore communities in 2015. The x-axis (MDS 1) of Fig 7 differentiated the coastal and Cashes Ledge sites offshore. The y-axis (MDS 2) primarily indicated differences between sites within regions, and in particular, separated the Spout Shoal site from all others. This was largely driven by the high percent cover of *D*. *japonica* at Spout Shoal (Table 3, S9 Table).

SIMPER analysis identified the species contributing significantly to the differences in benthic community structure between coastal and offshore sites. The high percent cover of invasive red algae, *D*. *japonica*, at the coastal sites contributed the most, nearly 20%, to the difference between coastal and offshore communities (S9 and S10 Tables). In contrast, the cover of the sediment / brown diatoms group was higher offshore, as were the coralline algal groups *Lithothamnion* sp., Corallinacea sp., and *Corallina officianalis*. A common pattern was that nearly all of the sessile invertebrates, including the sponge *Isodictya deichmannae*, hydroids, *Eudendrium* sp., mussels (*Modiolus modiolus*, *Mytilus edulis*), an encrusting bryozoan *Parasmittina jeffreysii*, and the ascidians *Aplidium constellatum*, *Mogula sp*., and *Diplosoma listeriatum* were more abundant at Cashes Ledge than at the coastal sites in 2015 and they contributed significantly to the onshore-offshore differences (S10 Table).

Analysis of spatio-temporal variation in benthic community structure at the Cashes Ledge sites (AR1 1987, AR1, AR 2) by nMDS, PERMANOVA and SIMPER revealed that they differed significantly in community composition (r^2^ = 0.31; p <0.001, S11 Table) and displayed 3 major patterns. First, the AR 1 1987 samples grouped together with the 2 other AR sites sampled in 2015 on the nMDS plot, suggesting that the characteristics that made the Cashes Ledge communities different from the coastal communities persisted over a 28-year period of warming in the Gulf of Maine (1987–2015), at least at the AR1 site (Fig 7, S11 Table). Secondly, there were significant changes in benthic community structure at the Ammen Rock 1 site between 1987 and 2015 (Fig 7, S11 Table). The Ammen Rock community in 2015 had nearly a 6-fold higher cover of the sediment / brown diatoms group, but depending on the specific AR site, the percent cover of 6 to 8 species of sessile invertebrates and 2 species of algae declined significantly between 1987 and 2015 (AR1 1987 vs AR 1 2014, AR 1 1987 vs AR2 2015, S11 Table). Finally, there were significant spatial differences between the macroalgal communities on Cashes Ledge (AR1, AR2 sites) as shown in the dispersion of quadrats in the mMDS plot and the SIMPER analysis (Fig 7, S11 Table). These differences were driven by a greater percent cover of *Chondrus crispus*, *D*. *japonica* and Corallinaceae at the AR 2 site (S11 Table).

### Fish

**Fig 8:**
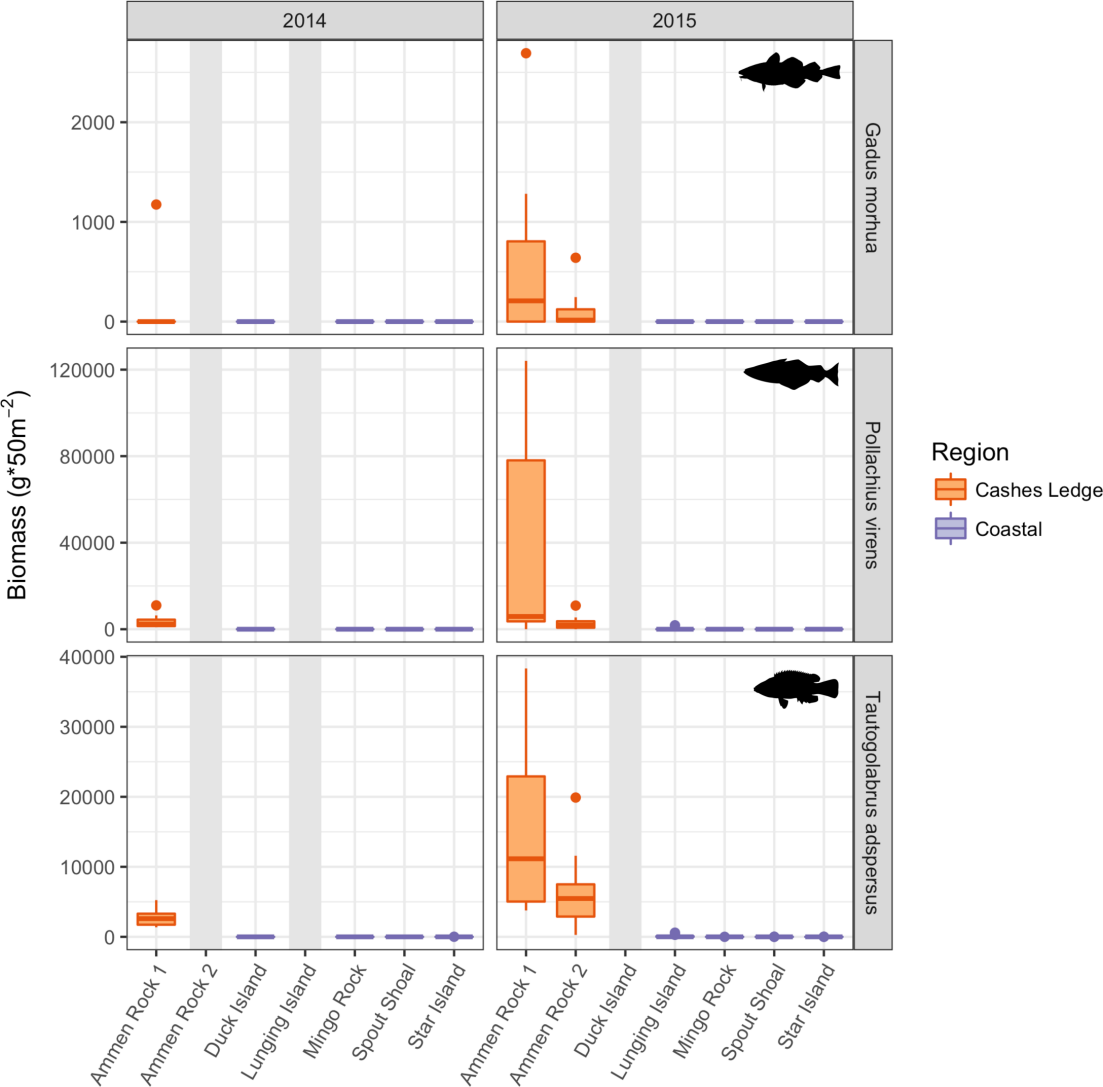
Fish biomass standardized to 10.0 x 5.0 m census areas for the three most abundant species: cod, pollock, cunner, comprising > 99.7% of total fish biomass. Grey bars indicate sites that were not visited in a specific year. Boxplots as in Fig 3. Note different scales of y-axes.

The mean total fish biomass per 50 m^2^ survey area ranged from 52,000 g (49,180 SD) on Cashes Ledge at AR1 in 2015 to 0 g at SS in the coastal zone in 2014. On average, total fish biomass at Cashes Ledge was 305 times greater than at coastal sites (Fig 8). The two-way ANOVA testing for differences between sites and years revealed that Cashes Ledge sites supported significantly higher total fish biomass than at all coastal sites (both 2014 and 2015, p < 0.001; S12 Table). There were no differences in fish biomass between AR1 and AR2, although AR1 was the only site to demonstrate significantly lower biomass in 2014 than in 2015 (p < 0.001). This may have been due to limited sampling at AR1 in 2014. Among coastal sites, the only significant difference in total fish biomass observed was between LI (283 g; 746 SD) and MR (5 g; 7 SD; p = 0.028, S12 Table).

A total of nine fish species were observed in the visual census transects (S12 Table). Pollock (*Pollachius virens*) yielded the highest biomass value of all species at 36,000 g per 50 m^2^ (50,800 SD) at AR1 in 2015, with a global average across all sites of 4,528 g. Cunner (*Tautogolabrus adspersus*) were the most numerous species, and also reaching peak biomass at AR1 in 2015 (15,420 g; 12,040 SD). Cod (*Gadus morhua*) were observed consistently but at low densities in transects at Cashes Ledge sites, where mean biomass ranged from 56 g (AR1 in 2015) to 113 g per 50 m^2^ (AR2 in 2015). No cod were observed in any transects at the coastal sites. All 6 other species (*Pholis gunnellus*, *Hemitripterus americanus*, *Zoarces americanus*, *Cyclopterus lumpus*, *Myoxocephalus* sp., and *Ulvaria subbifurcata*) were observed only sporadically and constituted less than 0.27% of total fish biomass.

**Fig 9:**
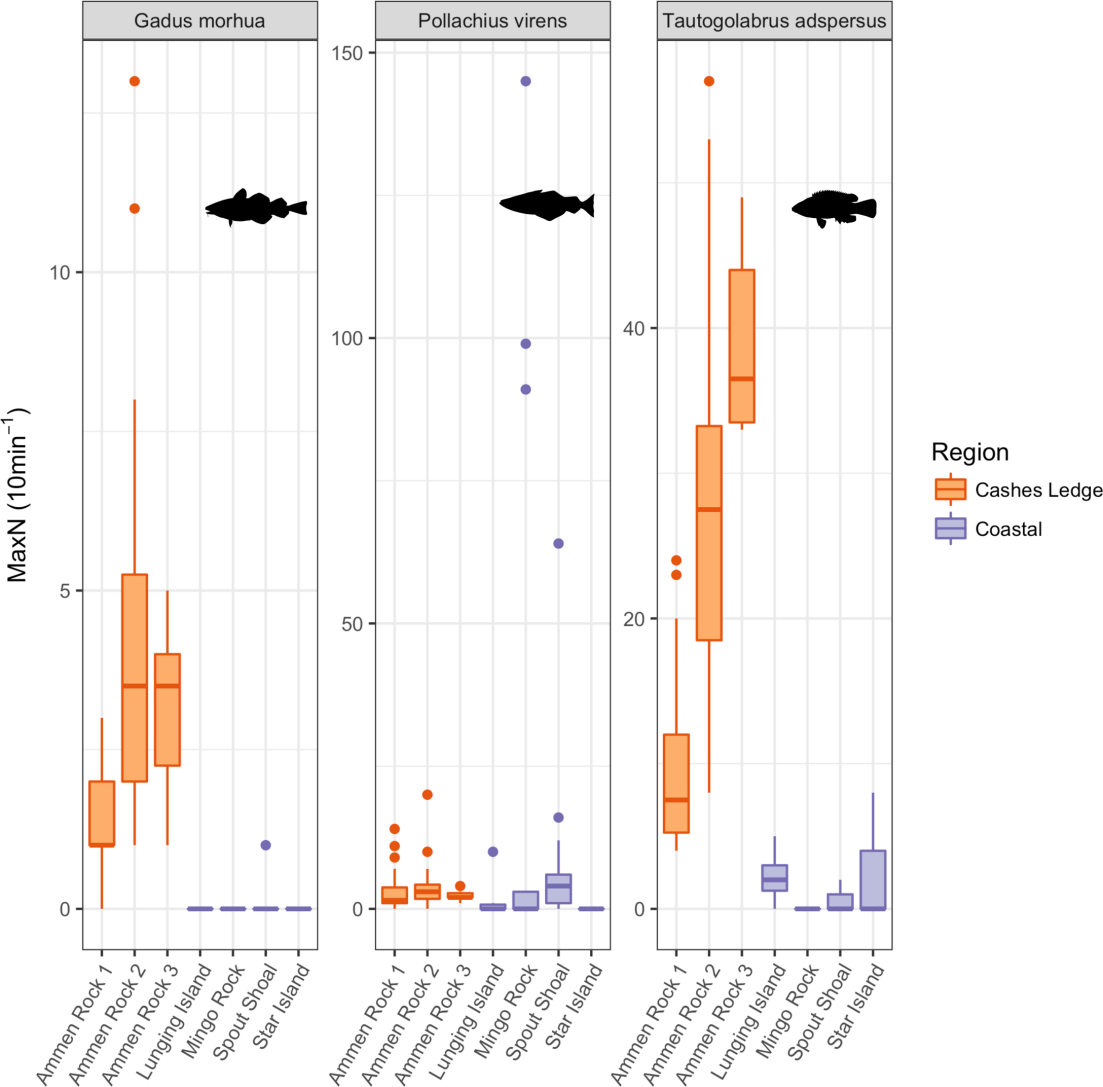
Abundance (maximum number of individuals observed in a single frame–MaxN–during a 10-minute segment) of the three fish species observed in stationary video deployments. Data are compiled for all years (2014–2016) to facilitate coastal to Cashes Ledge comparisons. Boxplots as in Fig 3. Note different scales on y-axes.

**Fig 10:**
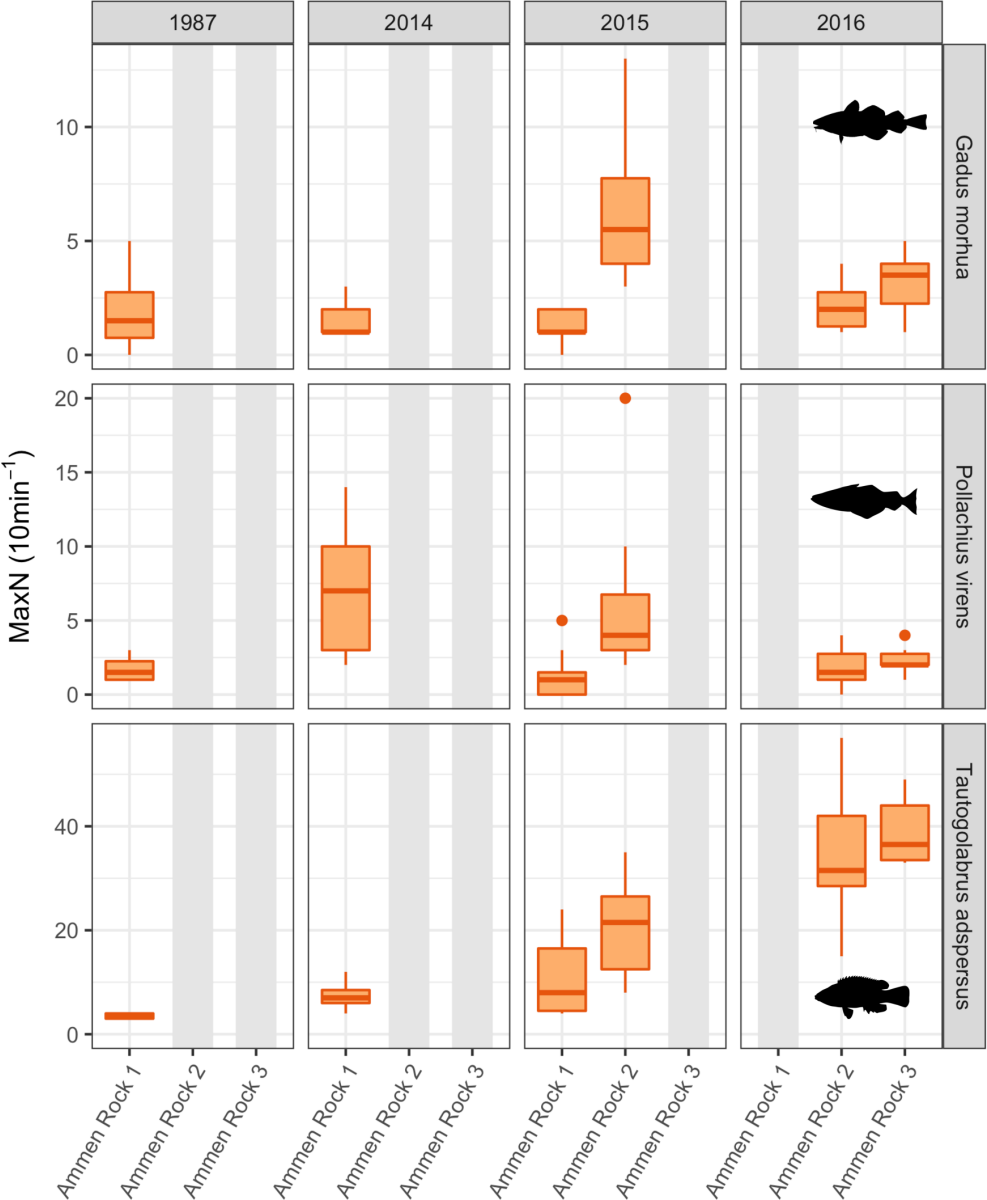
Temporal variation in fish abundance on Cashes Ledge 1987–2016. **Data shown are MaxN per 10-minute video segment** [54]. Coastal sites are not shown since data are only available for 2015. Boxplots as in Fig 3. Note different scales on y-axes.

Stationary video cameras were effective at detecting the three most common fish species: cod, pollock, and cunner (Figs 9 and 10, S1 Video). All three species displayed significant differences in abundance between sites (S13 Table). Cod abundance was highest at AR2 in 2015 (mean MaxN = 6.4; 3.4 SD), and cod were observed at all sites on Cashes Ledge during each sampling year (Figs 9 and 10). Only one individual cod was observed at any of the coastal sites (SS in 2015), yielding significantly higher abundance at all Cashes Ledge sites than at coastal sites (p < 0.001, S13 Table). Cunner ranged in abundance from 0 individuals at MR in 2015 and SI in 2014 to 39 individuals (6.9 SD) at AR3 in 2016. Overall, cunner averaged 20.96 individuals per 10-minute video segment at Cashes Ledge sites compared to 1.16 individuals at coastal sites, also a significant difference (p < 0.001; S13 Table). Among coastal sites, cunner were significantly more abundant at LI than at SS or MR (p = 0.0475; p < 0.001, respectively), and more abundant at SI than at MR (p = 0.017). Pollock were most common at MR in 2015 (20.29 individuals; 44.8 SD), and were only absent from SI. Pollock abundance was also significantly higher at SS than at LI, SI, and AR1 (p < 0.001; p < 0.001; p = 0.045, respectively; S13 Table).

At Cashes Ledge, at least one Ammen Rock site was sampled each year from 1987 to 2016 (Fig 10). A temporal comparison of the combined values from all three AR sites revealed that there were no significant differences between 2014–2016 in cod abundance (p = 0.364; S14 Table). Cunner were significantly more abundant at Cashes Ledge in 2016 than in 2015 or 2014 (p < 0.001). In contrast, pollock were significantly more abundant in 2014 than in 2015 or 2016 (p = 0.020; p = 0.016, respectively). Only one video was available from 1987, which showed approximately equal abundances of cod, but lower abundances of cunner and pollock than in 2014–2016. These data were not included in statistical analyses due to low sampling.

## Discussion

### Spatial variation in the Gulf of Maine

This study demonstrates that extreme spatial variation in shallow kelp forest communities occurs within the same depth range between coastal and offshore regions of the Gulf of Maine, an area of rapid ocean warming [60, 61] (Fig 2), underscoring the importance of incorporating regional variation into macroecological studies of population and community structure in general, and specifically for understanding kelp forest persistence. *Saccharina latissima* kelp dominates the high standing biomass forest at 12–15 m depth offshore at the Ammen Rock sites on Cashes Ledge, creating a dense, structurally complex habitat that, together with the associated invertebrate community, likely fosters the high population densities and biomasses of fish (cunner, cod, pollock) found associated with the *S*. *latissima* forest. Similarly, the abundance of epibenthic fish is positively associated with the presence of laminarian kelp in several bays in Maine USA [38]. Across the Atlantic, the species richness of the associated benthic invertebrate community is significantly related to the percent cover of *S*. *latissima* [3] and to *Laminaria hyperborea* [62].

The shallow kelp forest communities on Cashes Ledge represent an oasis of unusually high kelp and fish abundance in the Gulf of Maine, and as such, comprise an abundance hotspot that is likely functionally significant for sustained biological productivity of offshore regions of the GOM. For example, the *Saccharina latissima* kelp forest at the Ammen Rock sites on Cashes Ledge represent the highest average density and biomass values recorded at intermediate depth ranges (12–15 m) in the Western North Atlantic (S15 Table). High kelp biomass has been shown to support herbivore, filter feeder and detritivore components of food webs elsewhere [12, 63, 64]. We have observed many detached *S*. *latissima* kelp plants in deep sedimentary basins on the flanks Cashes Ledge (> 80 m) from submersibles (J. Witman *personal observations*), which suggests that drift kelp is supplied to consumers in deeper, adjacent habitats offshore. In addition, standardizing our fish transect data to individuals per 500 m^2^ for comparison to a global study [65] helped put the magnitude of the Cashes Ledge fish hotspot into perspective. It revealed that the average densities of all fish on Cashes Ledge (1227.5 individuals per 500 m^2^, S16 Table) was more than twice the average density of fish in the temperate western North Atlantic region surveyed by Stuart-Smith et al. [65] (586.7 individuals per 500 m ^2^).

The kelp forest at 12–15 m depth on Cashes Ledge is similar to the most abundant kelp forest living at half that depth in coastal regions of the NW Atlantic, emphasizing its unique status. For instance, comparisons of *S*. *latissima* abundance on Cashes Ledge to other studies indicated that similar peak densities to those at the AR 1 site (47.8 plants per m^2^, 1987) occurred at a substantially shallower depth of 6.0 m (45.0 and 60.0 plants per 1.0 m ^2^) off the central coast of Nova Scotia (Little Duck Island) in 1995 and 1997, respectively (S15 Table, [11]). Average biomasses comparable to the maximum value of 5.5 kg/ 1.0 m ^2^ at the AR 1 site in 2014 were also recorded from 6 m depth at Little Duck Island (5.8 kg/ 1.0 m ^2^) Nova Scotia (S15 Table, [11]). The intertidal-subtidal ecotone of Cobscook Bay, Maine supported densities of large *S*. *latissima* (> 50 cm length) ranging from 7.0 to 12.9 plants per m^2^ [66]. Kelp densities at two coastal sites were at least 50% lower than those at Ammen Rock Site 1 in 1987 (22.0 *S*. *latissima* plants per 1.0 m^2^ at 8 m depth, SI, 23.6 individuals per 1.0 m^2^, combined densities of *S*. *lattissima*, *S*.*digitata* at 10 m depth, Murray Rock, J. Witman *personal observations*) [67]. On Cashes Ledge, *Saccharina* sp. kelp densities decrease with depth, from intermediate (12–15 m) depths at the AR sites to 30 m at Ammen Rock Pinnacle, where densities were approximately 86% lower [27]. The consistent pattern of high *A*. *clathratum* densities at turbid coastal sites coupled with observations of a deep (> 30 m) *Agarum* dominated zone below a *Saccharina* kelp zone at Ammen Rock Pinnacle, Cashes Ledge [26, 27] suggests that *Agarum* is more tolerant to low light levels than *Saccharina* spp. Alternatively, *Agarum* is competitively inferior to other kelp species [68] and more resilient to urchin grazing than other kelp species [29, 68] indicating that interspecific competition and selective grazing may also contribute to the spatial differences in *A*.*clathratum* abundance documented in this study.

Cashes Ledge harbors denser fish populations with larger body sizes than found in comparable areas in the coastal Gulf of Maine (Fig 9) [65]. These results were confirmed by two methods, and by the time series of cod, pollock, and cunner populations over three years of video surveys (as well as qualitative comparison to 1987), substantiating consistently high abundances of all three species at Cashes Ledge. Given our concurrent observations of high kelp biomass, some of the differences in fish biomass between Cashes Ledge and the coastal zone are likely driven by the combination of greater three-dimensional habitat provided by the dense kelp canopy [37] and greater availability of kelp forest-associated food resources [40]. In particular, young cod in the northwest Atlantic prefer dense macroalgal beds for predator defense [69], as opposed to rock with more prostrate forms of macroalgae, such as at the coastal sites dominated by *A*. *clathratum* in our study. Pollock use intertidal habitats as juveniles at coastal locations, but are attracted to macroalgal beds for refuge from predation and to forage on benthic invertebrates [70]. This same type of habitat provision may occur at Ammen Rock, where kelp beds provide a key nursery habitat for recently settled fish.

### Temporal variation on Cashes Ledge

The time series spanning 28 years at Ammen Rock Site 1 revealed an overall trend of declining *S*. *latissima* densities, which likely released space from the competitively dominant *S*. *latissima* [55] and increased light below the canopy, favoring *S*. *digitata* kelp and invasive *D*. *japonica*, both of which increased significantly on Cashes Ledge over the study period. Interspecific competition for light is common between canopy forming kelp and understory algae [1]. An exceptional ocean heat wave during July–August 2012 [60] coincided with a sharp reduction in kelp densities in 2012 and increased encrustation of *S*. *latissima* kelp by the invasive bryozoan *Membranipora membranacea*. Since the growth of Atlantic laminarian kelp decreases above 15° C, and temperatures above 22.0 to 23.0° C degrees are lethal [46, 71], the exceptionally high surface temperatures during 2012 may have caused kelp mortality at the Ammen Rock sites. A mechanism exists to deliver warm surface waters to the benthos living as deep as 39 m in the form of internal waves, which frequently downwell the upper mixed layer on Cashes Ledge during the summer stratified season [35]. Encrustations of *M*. *membranacea* are known to increase the deterioration and dislodgement of *Saccharina* kelp [25, 72]. Consequently, we suggest that both global (increased temperature) and local (invasive species effects, interspecific competition among kelp species) factors acted synergistically to drive an overall decline in the foundation species *S*. *latissima* at the AR1 site from 1987 to 2015. While this may lead to an erosion of ecosystem services provided by *S*. *latissima* on Cashes Ledge, the loss may be partially offset by the increasing abundance of the understory kelp, *S*. *digitata* at AR2 between 2015 and 2016. High wave disturbance alone is an unlikely cause of the 2012 decline in *S*. *latissima* at AR1 since the 95% cumulative percentile of wave heights recorded in the Cashes Ledge area was the lowest wave height recorded during all years that kelp were surveyed (S2 Table). Our finding of consistently more abundant *Saccharina* kelp in warmer waters on Cashes Ledge than at cooler coastal sites is counterintuitive, as kelp growth and survival is limited at higher temperatures. However, the lower abundance and decline of kelp in the southwest GOM has been greatly influenced by sea urchin grazing and long term, negative impacts of invasive species [5, 10, 30].

The invasive red alga *Dasysiphonia japonica* has spread rapidly throughout the coastal, shallow subtidal zone of New England since its initial discovery in Rhode Island, USA in 2007 [58, 30]. It was absent from Cashes Ledge in 1987, appearing for the first time in the photo quadrats taken there in 2015, where it persisted into 2016. The highest cover (81.9%) recorded in this study from a coastal site (SS) at 12–15 m depth is comparable to the cover of *D*. *japonica* at 6 m depth off Nahant, MA, USA [58]. To our knowledge, this is the first record of *D*. *japonica* invasion into deeper, rocky subtidal habitats of the offshore region of the GOM. The significantly lower abundances of *D*. *japonica* on Cashes Ledge may result from 3 mechanisms that warrant testing; 1) limited dispersal of *D*. *japonica* from the coast to Cashes Ledge, 2) high herbivory on *D*. *japonica* on Cashes Ledge, or 3) competitive inhibition by *S*. *latissima*. If the last mechanism is supported, it implies that one of the ecosystem functions of *S*. *latissima* as a foundation species is to inhibit invasive species of algae in the understory. In other temperate subtidal ecosystems, herbivory can dampen the ability of subordinate algal species to increase after disturbance [73].

The significant shift in benthic community structure at the AR1 site on Cashes Ledge (Fig 7, S8 and S11 Tables) between 1987 and 2015 was characterized by a reduction in the cover of sessile invertebrates including *Parasmittina jeffreysii*, *Mytilus edulis*, *Aplidium constellatum*, *Isodictya deichmannae* and *Mogula* sp. and the red alga *Phycodrys fimbriata*. As there was a 5-fold increase in the cover of a matrix of sediment and diatoms over the same time period, the reduction in sessile invertebrate cover may have been caused by negative impacts of increased sedimentation on Cashes Ledge. Genovese and Witman [74] found that the abundance and survival of *P*. *jeffreysii* bryozoans was lower in coastal habitats with higher sedimentation than offshore on Cashes Ledge. Alternatively, both decreasing invertebrate and *Phycodrys* cover may reflect adverse effects of spatial competition with the red alga *D*. *japonica* which invaded Cashes Ledge sometime between 1987 and 2015.

#### Persistence in a warming Gulf of Maine

Although densities of kelp foundation species, cod and the cover of sessile invertebrates on Cashes Ledge have changed since the study began in 1987, extraordinary spatial differences in kelp forest community structure between coastal and offshore sites have persisted as the Gulf of Maine has warmed more rapidly than 99.9% of the world’s oceans [60]. Whether these characteristic differences and abundance hotspots on Cashes Ledge can persist in the face of future ocean warming is an important question, and clearly warrants future investigation with greater replication of long-term sites. However, we found evidence for persistence on three levels; in cod, kelp and in benthic community structure, and hypothesize that kelp and cod will respond synergistically to future warming of the Gulf of Maine.

While the spawning stock biomass of cod has decreased by an order of magnitude in the Gulf of Maine since the 1980’s [61, 75], the spatial pattern of higher cod abundance on Cashes Ledge than in the coastal GOM initially recorded from deep (30 m) rocky subtidal habitats during 1987–1990 [33, 76] has persisted for 25 to 28 years, at least at intermediate depths (12–15m, this study). That this spatial pattern persists, albeit with a lower density cod population, is remarkable, given that the rapid warming of the GOM since and continued overfishing have negatively impacted GOM cod [58, 61] (Fig 2). Cod and other GOM fishes are highly sensitive to temperature changes [61, 75], and both cod and pollock are projected to be negatively affected by long-term climate change [77–79]. Juvenile cod are positively associated with structurally complex habitats such as those created by macroalgae and heterogeneous hard substrate [69, 80, 81], which may increase their survival from predators [82]. Off Norway, the experimental removal of *Laminaria hyperborea* kelp led to a significant reduction in the number of small gadid fish compared to control areas with greater than 50% kelp cover [82]. We hypothesize that the ecological benefits (e.g., refuge from predation, availability of prey resources) of the kelp foundation species at the Ammen Rock sites on Cashes Ledge have had a positive effect on cod populations over the past several decades. This positive influence may occur despite northwards range shifts of GOM cod coincident with ocean warming over a similar period to our study [83]. It is likely that the closure of Cashes Ledge to ground fishing in 2002 [84] also helped maintain the pattern of higher cod abundance on Cashes than in the coastal GOM. Indeed, in a study of the life history of cod in and out of GOM fishery closures, Sherwood and Grabowski [43] found that cod inside the Cashes Ledge closed area, which includes the kelp forest in the Ammen Rock area, were significantly older and had a broader age structure and diet than cod outside of the Cashes closed area, suggesting a positive impact of the Cashes Ledge closure on cod populations.

Although the fundamental pattern of more abundant kelp on Cashes Ledge than at the same depths in the coastal SW GOM has persisted since 1987, *S*. *latissima* kelp densities declined from 1987–2015 at the one long-term site on Cashes Ledge (AR 1). A parallel trend of declining *Saccharina* kelp has been demonstrated in the coastal SW region of the GOM [12,30]. However, our ability to determine whether or not the trend of declining *S*. *latissima* kelp densities is typical of broader regions of the Gulf of Maine is limited, at present, by the lack of long term data from many other sites.

The occurrence of declining *S*. *latissima* kelp over two decades on Cashes Ledge (AR 1) does, however, raise the possibility that if the GOM continues to warm rapidly (Fig 2), [60, 61] both kelp and cod will decline independently as their physiological temperature optima are exceeded, and synergistically, by a negative feedback initiated by declining kelp cover. In this scenario, as the *S*. *latissima* kelp forest continues to thin on Cashes Ledge, there will be less protective cover and food resources for juvenile cod, increasing their mortality. Future research is needed to monitor the thermal regime and its effects on kelp and cod populations on Cashes Ledge in order to evaluate their co-dependence, resilience and sustainability. The importance of understanding the link between kelp and fish populations on Cashes Ledge is underscored by a review of the effects of kelp on fisheries which indicated that 67% of all studies found a positive relationship between kelp and fishery variables [85]. Fish, kelp and benthic communities on Cashes Ledge may be particularly vulnerable to ocean warming given that SST in the vicinity of Cashes Ledge is higher than in the coastal zone (S1 and S2 Figs).

Since storm disturbance increases kelp mortality [7, 86–88] and the frequency of extreme storms is predicted to increase with global warming [88, 89], increasing wave disturbance as well as warmer temperatures may also contribute to the future loss of kelp foundation species in the Atlantic [8]. In this study, the northern areas of the coastal and Cashes Ledge sites were both extremely exposed to high wave action (S3 Fig, S2 Table). Greater wave heights in the Cashes Ledge region suggest that wave disturbance plays a larger role in structuring kelp forests there than at the coastal sites.

The last example of persistence was seen at the community level. Even though there was a shift in the species composition of the benthic community at the AR 1 site between 1987 and 2015, the 1987 samples still grouped together with the other AR sites sampled in 2015 in the nMDS analysis, indicating that the characteristics that made the Cashes Ledge communities distinct persisted over a 28-year period of warming in the Gulf of Maine (1987–2015, Fig 7, S11 Table). Greater dispersal of species between coastal and offshore regions, further invasion of offshore communities by introduced species [89], negative impacts of warming on offshore populations, and overfishing of cod offshore will tend to homogenize the striking differences documented here between coastal and offshore kelp forest communities in the Gulf of Maine.

### Implications for trophic structure

Given the near absence of kelp-consuming sea urchins in the shallow, offshore kelp forests on Cashes Ledge and their abundant presence at coastal sites, a logical question is: to what extent do the patterns of higher kelp abundance offshore reflect a reduction in urchin herbivory vs differences in other biological or environmental drivers affecting kelp between coastal and offshore regions of the GOM? In terms of environmental drivers, greater light penetration to depth offshore was ranked as a leading factor accounting for the occurrence of unusually deep (30 m) *Saccharina* kelp assemblages at Ammen Rock Pinnacle on Cashes Ledge [26]. It is plausible that greater light penetration offshore is also a definitive factor for high kelp abundance at shallower depths on Cashes Ledge (this study). The scarcity of *S*. *droebachiensis* urchins in deeper (30 m) Cashes Ledge communities was attributed to higher fish predation offshore, as wolffish (*Anarachias lupus*) readily consumed large (7.0–8.0 cm TD) tethered sea urchins at two sites on Cashes Ledge but not at coastal GOM sites [76], and the abundance of fish known to prey on sea urchins was higher offshore [33, 76]. Vadas and Steneck [76] compared the attack rates on tethered small (2.0 cm TD), intermediate (5.0 cm) and large urchins in logistically difficult experiments performed at deep coastal sites with low predatory fish abundance versus deep sites on Cashes Ledge where predatory fish were abundant. However, their results showed considerable overlap of 95% confidence intervals with the mean attack rates on urchins in the 2 groups (low vs high fish abundance, Fig 4 in [76]) which hampers firm conclusions about differences in the magnitude of fish predation on urchins. Previous studies in deep Cashes Ledge communities found higher fish (cod, wolfish) predation on rock crabs (*Cancer* sp.) than at deep coastal GOM sites, and that crabs were scarce offshore [33]. Both studies [33, 76] invoked a higher degree of top down control offshore on Cashes Ledge than in the coastal GOM, as crabs eat kelp-consuming sea urchins [90–93] among other invertebrate prey, while large predatory fish have the potential to regulate sea urchin populations, releasing kelp from herbivory. The greater abundance of crab prey (mussels) at Ammen Rock versus the coastal sites, may reflect a release from crab predation offshore or simply, unique patterns of mussel recruitment on Cashes Ledge.

Our results on coastal–offshore differences in the abundance of small sea urchins strongly suggest that sea urchin populations are limited by a near failure of recruitment over multiple years to the shallow offshore kelp forests on Cashes Ledge. There isn’t enough information presently to discriminate between supply-sided (i.e. lack of urchin larval dispersal and/or larval settlement to Cashes Ledge) or post-settlement (i.e. high predation on settling and/or juvenile urchins) processes as the cause of low urchin densities on Cashes Ledge. Rigorous tests of recruitment limitation of these important consumers (urchins, crabs) on Cashes Ledge, either by dispersal failure or post-settlement predation, need to be performed as alternate hypotheses to that of top-down population control. In particular, post-settlement predation on small urchins by cunner warrants attention as a possible mechanism regulating urchin populations in the shallow Cashes Ledge kelp forest because cunner are significant predators of small urchins [5, 94]. Furthermore, cunner population densities were significantly higher at 12–15 m depths on Cashes Ledge than at the coastal sites, and their populations have increased since 1987, and significantly from 2014–2016 at the Ammen Rock sites (Fig 10). Consequently, cunner predation may have an influential effect on the distribution and abundance of small prey, such as young of the year and juvenile sea urchins in the shallow Cashes Ledge kelp forest.

## Conclusions

This study highlights the need to incorporate regional scale variation into studies of marine community dynamics and into investigations of the ecological effects of climatic forcing. Striking differences in every component of the kelp forest community analyzed (kelp, fish, benthic invertebrates, understory algae) occurred between coastal and offshore sites at the same depth in the Gulf of Maine, characterizing Cashes Ledge as an abundance hotspot for *Saccharina latissima* kelp, cod, cunner and sessile invertebrates. Despite declines in the primary foundation species on Cashes Ledge, the kelp *S*. *latissima*, in association with local (effects of invasive bryozoans, potential competition with *S*. *digitata*) and global (ocean warming) stressors, the pattern of higher kelp abundance offshore persisted for nearly three decades, likely facilitating the maintenance of high fish abundance there, a hypothesis to be tested. The generally higher sea surface temperatures in the Cashes Ledge area than at the northern extent of the coastal sites suggests that the Cashes Ledge marine ecosystem provides an important opportunity to study the effects of global warming in the Gulf of Maine.

## Supporting information

S1 Fig Regional temperature differences between coastal (Western Maine Shelf; WMS) and offshore (Cashes Ledge; CL) buoy locations. Temperatures are derived from NOAA oceanographic buoys at 1m depth.
(PDF)

S2 Fig Temperatures during an anomalously warm year (2012) from Western Maine Shelf and Cashes Ledge buoys. Temperatures are derived from NOAA oceanographic buoys at 1m depth.
(PDF)

S3 Fig Cumulative distributions of significant wave heights, WMS vs CL. Wave heights are derived from NOAA oceanographic buoys at 1m depth.
(PDF)

S1 Table 95 and 99^th^ percentile Temperatures, WMS vs CL. Temperatures are derived from NOAA oceanographic buoys at 1m depth.
(PDF)

S2 Table 95 and 99^th^ percentile significant wave heights, WMS vs CL. Wave heights are derived from NOAA oceanographic buoys at 1m depth.
(PDF)

S3 Table ANOVA and multiple comparison tests on kelp biomass. Data are derived from fresh-weight measurements of all kelp individuals collected within 1.0 m^2^ quadrats. One-way ANOVA tests were used to examine differences between sites for biomass of each species of kelp in each year, followed by Tukey’s Honest Significant Difference tests for pairwise comparisons. We used the same procedure to test for the effect of year on kelp biomass at Ammen Rock 1.
(PDF)

S4 Table ANOVA and multiple comparison tests on kelp density. All data are derived from field counts of individual kelp stipes in 1.0 m^2^ quadrats. We performed individual one-way ANOVA tests for each species and year for the effect of site on kelp density. We used the same procedure to test for the effect of year on kelp biomass at Ammen Rock 1.
(PDF)

S5 Table Percent cover of invasive bryozoan, *Membranipora membranacea* on kelp at Cashes Ledge. Data are means with standard deviations (SD).
(PDF)

S6 Table ANOVA on percent cover of invasive red alga *Dasysiphonia japonica*. All data are derived from random-dot counts of *D*. *japonica* percent cover from 0.25 m2 quadrat photos. ANOVA was used to test the effect of site, followed by Tukey’s Honest Significant Difference test for pairwise comparisons.
(CSV)

S7 Table Densities of sea urchins *Stronglyocentrotus droebachiensis*. Data are means with Standard Errors (SE). Sample sizes are numbers of 1.0 m 2 quadrats searched. No sea urchins were found at the Ammen Rock sites 1 in 1987, 2012 and at Ammen Rock sites 2 and 3 in 2016 (n = 102, 1.0 m 2 total quadrats sampled at these sites).
(PDF)

S8 Table PERMANOVA of benthic species composition across all sites. We used the adonis() routine in the R package vegan to test for significant differences in multivariate species composition among all sites sampled in photo quadrat surveys taken in 2015.
(XLSX)

S9 Table SIMPER analysis of coastal vs offshore benthic species composition. Analysis indicates the contribution of each species or group to coastal vs offshore differences.
(XLSX)

S10 Table SIMPER analysis of benthic species composition across all sites. Analysis indicates the contribution of each species or group to between site differences of benthic communities sampled in photo quadrats taken in 2015.
(XLSX)

S11 Table PERMANOVA of benthic species composition at Cashes Ledge (Ammen Rock Sites) followed by SIMPER analysis. Analysis as in S8 and S9 Tables, comparing species composition at all Ammen Rock sites from photo quadrats taken in 1987 and 2015.
(XLSX)

S12 Table Temporal and spatial comparison of total fish biomass, followed by post- hoc tests. Data used are biomass (g/50m2). We performed a two-way ANOVA to test for significant differences between sites and years and their interaction, followed by Tukey’s Honest Significant Difference test for pairwise comparisons.
(PDF)

S13 Table Spatial comparison of fish abundance (cod, cunner, and pollock) by site, followed by post-hoc tests. Data used are MaxN: maximum number of individuals per species observed in a 10-minute segment of stationary video. For each species, we performed an ANOVA test for significant differences between sites, followed by Tukey’s Honest Significant Difference test for pairwise comparisons.
(PDF)

S14 Table Temporal comparison of fish abundance (cod, cunner, and pollock) by year (2014, 2015, and 2016) at Cashes Ledge sites, followed by post-hoc tests. Data used are MaxN: maximum number of individuals per species observed in a 10-minute segment of stationary video. For each species, we performed an ANOVA test for significant differences between years, followed by Tukey’s Honest Significant Difference test for pairwise comparisons.
(PDF)

S15 Table Comparison of kelp biomass and density at sites in the North West Atlantic. We compared kelp density and biomass on Cashes Ledge to kelp abundance within the same depth range (12–15 m) and kelp abundance at shallower depths (6–10 m) at other sites in the NW Atlantic. Multiple entries from the same site are from different sampling dates. *Saccharina longicruris = S*. *latissima; Laminaria digitata = S*. *digitata*. Data are from literature review: Krumhansl et al. 2016, and this study.
(PDF)

S16 Table Comparison of fish densities to results from global surveys of Stuart Smith et al. 2013. This analysis was performed on our UVC data standardized to 500m2 for comparison with other sampling efforts in the region (Stuart-Smith et al., 2013). Fish density at Cashes Ledge averaged 1227.5 (246.6 SE) individuals per 500m2, compared to only 24.8 (7.1 SE) individuals at coastal sites. Densities at Cashes Ledge were more than twice the average reported for the entire North Atlantic, whereas values at our coastal sites were less that 5% of the regional average (Stuart-Smith et al. 2013).
(PDF)

S1 Video Representative fish communities (cod, cunner, pollock) in the kelp forest at Ammen Rock, Cashes Ledge. Video by Evan Kovacs.
(MOV)
