# Supplementary figures and images for: Persistent differences between coastal and offshore kelp forest communities in a warming Gulf of Maine
Source: PLoS One. 2018 Jan 3;13(1):e0189388. doi: 10.1371/journal.pone.0189388 (PMC5751975; doi:10.1371/journal.pone.0189388)

Cashes Ledge

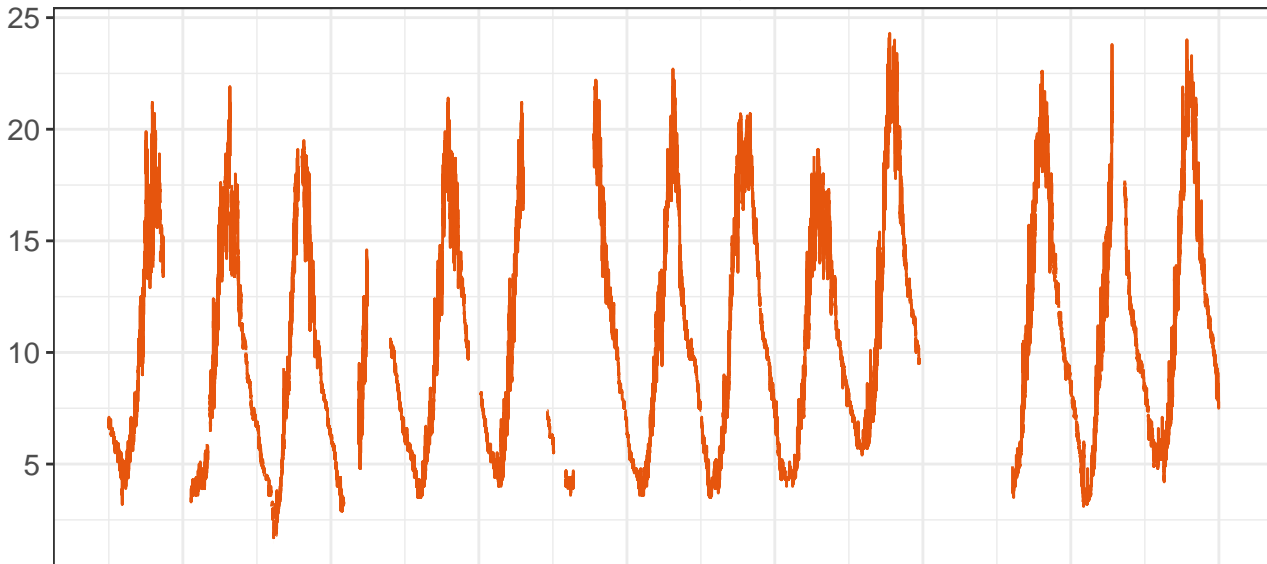

Western Maine Shelf

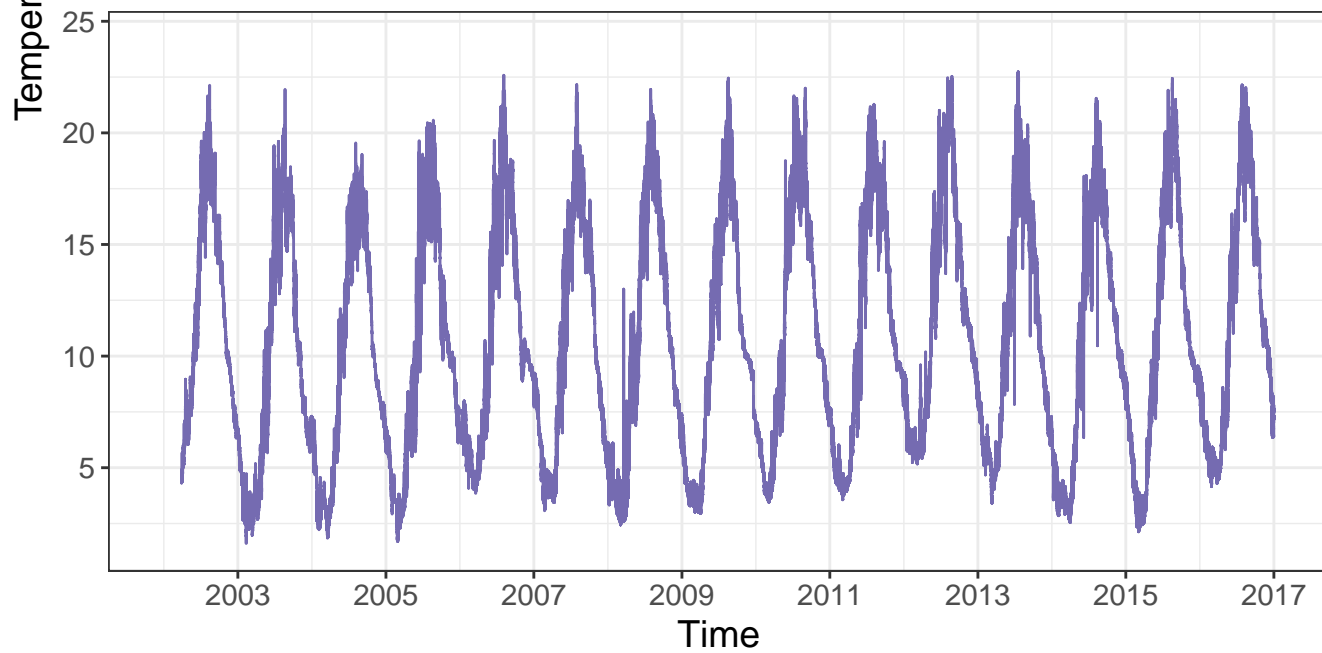

Supplement: S1 Fig — Temperatures are derived from NOAA oceanographic buoys at 1m depth. (PDF) [file pone.0189388.s001.pdf]

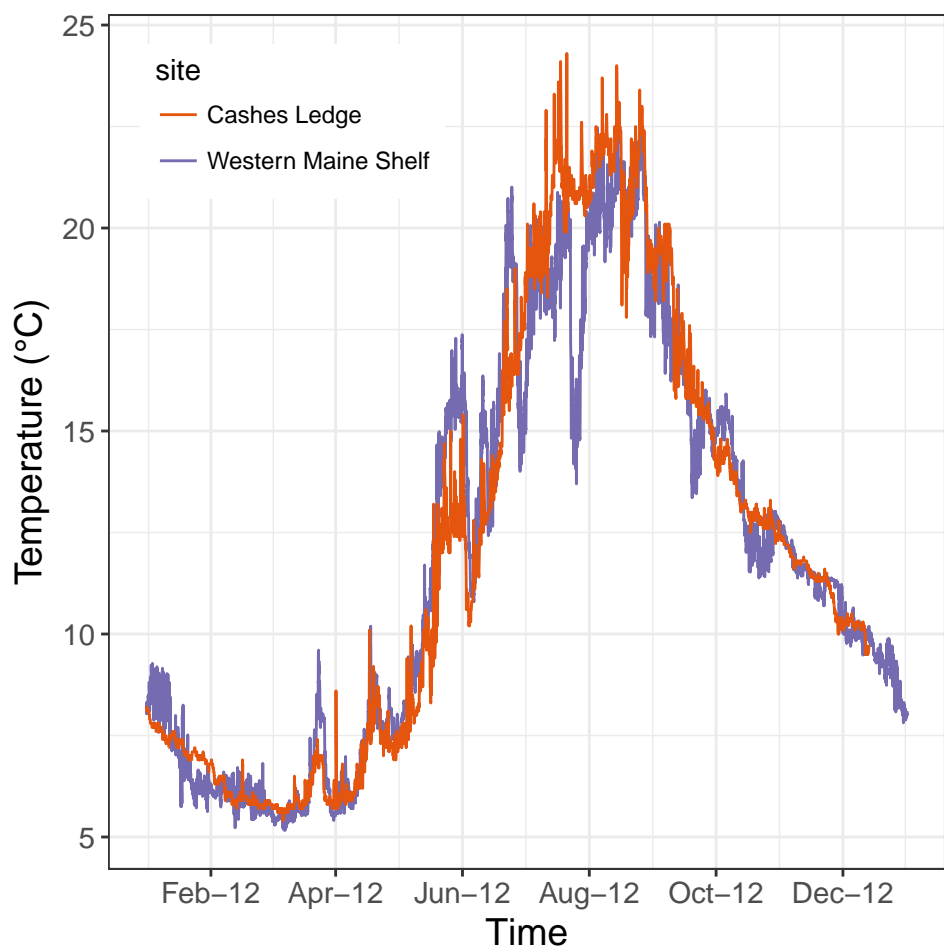

Supplement: S2 Fig — Temperatures are derived from NOAA oceanographic buoys at 1m depth. (PDF) [file pone.0189388.s002.pdf]

Significant Wave Height (m)

1987

2012

2014

2015

2016

10.0  
7.5  
5.0  
2.5  
0.0

0.25 0.50 0.65 0.85 1.00

0.25 0.50 0.65 0.85 1.00

0.25 0.50 0.65 0.85 1.00

0.25 0.50 0.65 0.85 1.00

0.25 0.50 0.65 0.85 1.00

Cumulative distribution

site

Cashes Ledge

Western Maine Shelf

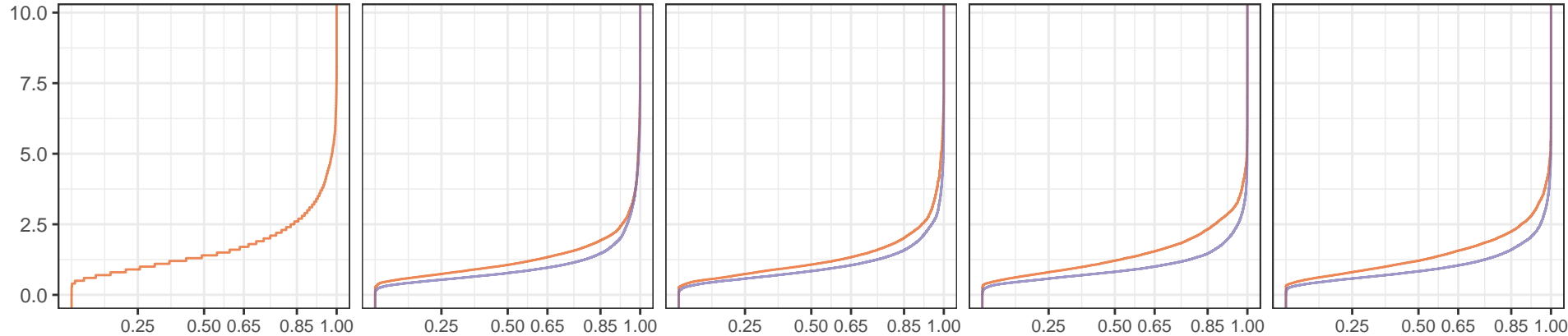

Supplement: S3 Fig — Wave heights are derived from NOAA oceanographic buoys at 1m depth. (PDF) [file pone.0189388.s003.pdf]
