# Supplementary material for: Persistent differences between coastal and offshore kelp forest communities in a warming Gulf of Maine
Source: PLoS One. 2018 Jan 3;13(1):e0189388. doi: 10.1371/journal.pone.0189388 (PMC5751975; doi:10.1371/journal.pone.0189388)
Supplement: S1 Table — Temperatures are derived from NOAA oceanographic buoys at 1m depth. (PDF) [file pone.0189388.s004.pdf]

S1 Table Comparison of 95 and 99 cumulative percentiles of annual sea surface temperatures (^{o}C, 1 m depth) on Cashes Ledge and in the Coastal Zone (Western Maine Shelf). Data are from buoy 44005 on Cashes Ledge (43 ^{o} 12'3" N, 69 ^{o} 7'42" W) and from Western Maine Shelf buoy station 44030 (43 ^{o} 10'51" N, 70 ^{o} 25'40" W). No data were available from the Western Maine Shelf buoy in 1987.

**Site**	**Year**	**95th**	**99th**
Cashes Ledge	1987	17.900	19.000
Cashes Ledge	2012	21.800	22.500
Cashes Ledge	2014	20.000	21.400
Cashes Ledge	2015	16.700	17.900
Cashes Ledge	2016	21.400	22.300
Western Maine Shelf	2012	20.536	21.512
Western Maine Shelf	2014	18.640	20.270
Western Maine Shelf	2015	19.640	20.670
Western Maine Shelf	2016	19.670	20.760
