# Supplementary material for: Persistent differences between coastal and offshore kelp forest communities in a warming Gulf of Maine
Source: PLoS One. 2018 Jan 3;13(1):e0189388. doi: 10.1371/journal.pone.0189388 (PMC5751975; doi:10.1371/journal.pone.0189388)
Supplement: S2 Table — Wave heights are derived from NOAA oceanographic buoys at 1m depth. (PDF) [file pone.0189388.s005.pdf]

S2 Table Comparison of 95 and 99 cumulative percentiles of annual significant wave heights (in meters) on Cashes Ledge and in the Coastal Zone (Western Maine Shelf). Data are from buoy 44005 on Cashes Ledge in 180 m water depth and from Western Maine Shelf buoy station 44030 in 62 m water depth (locations in S1 Table). Significant wave height represents the average of the highest one-third of all of the wave heights recorded during 20-minute sampling period. No data were available from the Western Maine Shelf buoy in 1987.

Site	Year	95th	99th
Cashes Ledge	1987	3.805	5.500
Cashes Ledge	2012	2.750	4.574
Cashes Ledge	2014	2.950	4.974
Cashes Ledge	2015	3.100	4.254
Cashes Ledge	2016	3.170	4.290
Western Maine Shelf	2012	2.498	4.396
Western Maine Shelf	2014	2.438	3.695
Western Maine Shelf	2015	2.276	3.343
Western Maine Shelf	2016	2.334	3.568
