# Supplementary material for: Persistent differences between coastal and offshore kelp forest communities in a warming Gulf of Maine
Source: PLoS One. 2018 Jan 3;13(1):e0189388. doi: 10.1371/journal.pone.0189388 (PMC5751975; doi:10.1371/journal.pone.0189388)
Supplement: S3 Table — Data are derived from fresh-weight measurements of all kelp individuals collected within 1.0 m2 quadrats. One-way ANOVA tests were used to examine differences between sites for biomass of each species of kelp in each year, followed by Tukey’s Honest Significant Difference tests for pairwise comparisons. We used the same procedure to test for the effect of year on kelp biomass at Ammen Rock 1. (PDF) [file pone.0189388.s006.pdf]

S 3 Table ANOVA and multiple comparison tests on kelp biomass. Data are derived from fresh-weight measurements of all kelp individuals collected within 1.0 m^2 quadrats. One-way ANOVA tests were used to examine differences between sites for biomass of each species of kelp in each year, followed by Tukey's Honest Significant Difference tests for pairwise comparisons. We used the same procedure to test for the effect of year on kelp biomass at Ammen Rock 1.

One-way ANOVA Factor: Site
Response Variable: 2014 A. clathratum biomass per 1.0 m2 (log(x + 1.1) transformed)

ANOVA Table

	Df	Sum of Squares	Mean Square	F for Model	Pr(>F)
Site	3	118.984	39.661	83.148	<.001
Residuals	21	10.017	0.477		

Tukey Multiple Comparison of Means

Site Comparison	Mean Difference	P-value (adjusted)
Lunging Island-Ammen Rock 1	6.021	<.001
Spout Shoal-Ammen Rock 1	3.528	<.001
Star Island-Ammen Rock 1	4.508	<.001
Spout Shoal-Lunging Island	-2.493	<.001
Star Island-Lunging Island	-1.514	0.008
Star Island-Spout Shoal	0.980	0.139

One-way ANOVA Factor: Site
Response Variable: 2014 S. digitata biomass per 1.0 m2 (log(x + 1.1) transformed)

ANOVA Table

	Df	Sum of Squares	Mean Square	F for Model	Pr(>F)
Site	3	<.001	<.001	1.400	0.271
Residuals	21	<.001	<.001		

One-way ANOVA Factor: Site
Response Variable: 2014 S. latissima biomass per 1.0 m2 (log(x + 1.1) transformed)

ANOVA Table

	Df	Sum of Squares	Mean Square	F for Model	Pr(>F)
Site	3	266.000	88.600	196.040	<.001
Residuals	21	9.490	0.452		

Tukey Multiple Comparison of Means

Site Comparison	Mean Difference	P-value (adjusted)
Lunging Island-Ammen Rock 1	-8.488	<.001
Spout Shoal-Ammen Rock 1	-7.982	<.001
Star Island-Ammen Rock 1	-7.448	<.001
Spout Shoal-Lunging Island	0.506	0.459
Star Island-Lunging Island	1.040	0.077
Star Island-Spout Shoal	0.534	0.593

One-way ANOVA Factor: Site
Response Variable: 2015 A. clathratum biomass per 1.0 m2 (log(x + 1.1) transformed)

ANOVA Table

	Df	Sum of Squares	Mean Square	F for Model	Pr(>F)
Site	5	191.000	38.300	127.120	<.001
Residuals	23	6.930	0.301		

Tukey Multiple Comparison of Means

Site Comparison	Mean Difference	P-value (adjusted)
Ammen Rock 2-Ammen Rock 1	0.483	0.776
Lunging Island-Ammen Rock 1	6.293	<.001
Mingo Rock-Ammen Rock 1	5.918	<.001
Spout Shoal-Ammen Rock 1	5.656	<.001
Star Island-Ammen Rock 1	5.211	<.001
Lunging Island-Ammen Rock 2	5.810	<.001
Mingo Rock-Ammen Rock 2	5.435	<.001
Spout Shoal-Ammen Rock 2	5.174	<.001
Star Island-Ammen Rock 2	4.728	<.001
Mingo Rock-Lunging Island	-0.375	0.884
Spout Shoal-Lunging Island	-0.637	0.465
Star Island-Lunging Island	-1.082	0.049
Spout Shoal-Mingo Rock	-0.262	0.972
Star Island-Mingo Rock	-0.707	0.354
Star Island-Spout Shoal	-0.445	0.791

One-way ANOVA Factor: Site
Response Variable: 2015 S. digitata biomass per 1.0 m2 (log(x + 1.1) transformed)

ANOVA Table

	Df	Sum of Squares	Mean Square	F for Model	Pr(>F)
Site	5	72.700	14.500	5.954	0.001
Residuals	23	56.100	2.440		

Tukey Multiple Comparison of Means

Site Comparison	Mean Difference	P-value (adjusted)
Ammen Rock 2-Ammen Rock 1	-4.141	0.007
Lunging Island-Ammen Rock 1	-4.141	0.007
Mingo Rock-Ammen Rock 1	-3.637	0.022
Spout Shoal-Ammen Rock 1	-1.262	0.831
Star Island-Ammen Rock 1	-4.141	0.007
Lunging Island-Ammen Rock 2	<.001	1.000
Mingo Rock-Ammen Rock 2	0.504	0.995
Spout Shoal-Ammen Rock 2	2.880	0.074
Star Island-Ammen Rock 2	<.001	1.000
Mingo Rock-Lunging Island	0.504	0.995
Spout Shoal-Lunging Island	2.880	0.074
Star Island-Lunging Island	<.001	1.000
Spout Shoal-Mingo Rock	2.375	0.196
Star Island-Mingo Rock	-0.504	0.995
Star Island-Spout Shoal	-2.880	0.074

One-way ANOVA Factor: Site
Response Variable: 2015 S. latissima biomass per 1.0 m2 (log(x + 1.1) transformed)

ANOVA Table

	Df	Sum of Squares	Mean Square	F for Model	Pr(>F)
Site	5	269.000	53.900	32.374	<.001
Residuals	23	38.300	1.660		

Tukey Multiple Comparison of Means

Site Comparison	Mean Difference	P-value (adjusted)
Ammen Rock 2-Ammen Rock 1	-0.347	0.998
Lunging Island-Ammen Rock 1	-6.382	<.001
Mingo Rock-Ammen Rock 1	-5.466	<.001
Spout Shoal-Ammen Rock 1	-6.631	<.001
Star Island-Ammen Rock 1	-7.886	<.001
Lunging Island-Ammen Rock 2	-6.035	<.001

Mingo Rock-Ammen Rock 2	-5.119	<.001
Spout Shoal-Ammen Rock 2	-6.285	<.001
Star Island-Ammen Rock 2	-7.539	<.001
Mingo Rock-Lunging Island	0.916	0.867
Spout Shoal-Lunging Island	-0.250	1.000
Star Island-Lunging Island	-1.504	0.459
Spout Shoal-Mingo Rock	-1.165	0.710
Star Island-Mingo Rock	-2.420	0.067
Star Island-Spout Shoal	-1.255	0.645

One-way ANOVA Factor: Site
Response Variable: 2016 A. clathratum biomass per 1.0 m2 (log(x + 1.1) transformed)

ANOVA Table

	Df	Sum of Squares	Mean Square	F for Model	Pr(>F)
Site	1	<.001	<.001	1.000	0.331
Residuals	18	<.001	<.001		

One-way ANOVA Factor: Site
Response Variable: 2016 S. digitata biomass per 1.0 m2 (log(x + 1.1) transformed)

ANOVA Table

	Df	Sum of Squares	Mean Square	F for Model	Pr(>F)
Site	1	15.500	15.500	1.743	0.203
Residuals	18	16<.001	8.900		

One-way ANOVA Factor: Site
Response Variable: 2016 S. latissima biomass per 1.0 m2 (log(x + 1.1) transformed)

ANOVA Table

	Df	Sum of Squares	Mean Square	F for Model	Pr(>F)
Site	1	2.640	2.640	2.893	0.106
Residuals	18	16.400	0.911		

Ammen Rock 1 temporal comparison

One-way ANOVA Factor: Year
Response Variable: A. clathratum biomass per 1.0 m2 (log(x + 1.1) transformed)

ANOVA Table

	Df	Sum of Squares	Mean Square	F for Model	Pr(>F)
Year	2	<.001	<.001		
Residuals	12	<.001	<.001		

One-way ANOVA Factor: Year
Response Variable: S. digitata biomass per 1.0 m2 (log(x + 1.1) transformed)

ANOVA Table

	Df	Sum of Squares	Mean Square	F for Model	Pr(>F)
Year	2	50.300	25.200	12.936	0.001
Residuals	12	23.300	1.940		

Tukey Multiple Comparison of Means

Year Comparison	Mean Difference	P-value (adjusted)
2014-2012	<.001	1.000
2015-2012	4.140	0.002
2015-2014	4.140	0.002

One-way ANOVA Factor: Year
Response Variable: S. latissima biomass per 1.0 m2 (log(x + 1.1) transformed)

ANOVA Table

	Df	Sum of Squares	Mean Square	F for Model	Pr(>F)
Year	2	3.720	1.860	5.653	0.019
Residuals	12	3.950	0.329		

Tukey Multiple Comparison of Means

Year Comparison	Mean Difference	P-value (adjusted)
2014-2012	1.060	0.025
2015-2012	0.948	0.060
2015-2014	-0.116	0.951
