# Supplementary material for: Persistent differences between coastal and offshore kelp forest communities in a warming Gulf of Maine
Source: PLoS One. 2018 Jan 3;13(1):e0189388. doi: 10.1371/journal.pone.0189388 (PMC5751975; doi:10.1371/journal.pone.0189388)
Supplement: S4 Table — All data are derived from field counts of individual kelp stipes in 1.0 m2 quadrats. We performed individual one-way ANOVA tests for each species and year for the effect of site on kelp density. We used the same procedure to test for the effect of year on kelp biomass at Ammen Rock 1. (PDF) [file pone.0189388.s007.pdf]

S4 Table. ANOVA and multiple comparison tests on kelp density. All data are derived from field counts of individual kelp stipes in 1.0 m^2 quadrats. We performed individual one-way ANOVA tests for each species and year for the effect of site on kelp density. We used the same procedure to test for the effect of year on kelp biomass at Ammen Rock 1.

One-way ANOVA Factor: Site
Response Variable: 2016 S. latissima density per 1.0 m2 (log(x + 1.1) transformed)

ANOVA Table

	**Df**	**Sum of Squares**	**Mean Square**	**F for Model**	**Pr(>F)**
Site	1	0.988	0.988	1.726	0.205
Residuals	18	10.300	0.572		

One-way ANOVA Factor: Site
Response Variable: 2016 S. digitata density per 1.0 m2 (log(x + 1.1) transformed)

ANOVA Table

	**Df**	**Sum of Squares**	**Mean Square**	**F for Model**	**Pr(>F)**
Site	1	8.340	8.340	4.930	0.039
Residuals	18	30.400	1.690		

One-way ANOVA Factor: Site
Response Variable: 2016 A. clathratum density per 1.0 m2 (log(x + 1.1) transformed)

ANOVA Table

	**Df**	**Sum of Squares**	**Mean Square**	**F for Model**	**Pr(>F)**
Site	1	0.000	0.000	1.000	0.331
Residuals	18	0.000	0.000		

One-way ANOVA Factor: Site
Response Variable: 2015 S. latissima density per 1.0 m2 (log(x + 1.1) transformed)

ANOVA Table

	**Df**	**Sum of Squares**	**Mean Square**	**F for Model**	**Pr(>F)**
Site	5	101.000	20.200	142.570	<.001
Residuals	47	6.650	0.142		

Tukey Multiple Comparison of Means

Site Comparison	Mean Difference	P-value (adjusted)
Ammen Rock 2-Ammen Rock 1	0.124	0.983
Lunging Island-Ammen Rock 1	-2.582	0.000
Mingo Rock-Ammen Rock 1	-2.336	0.000
Spout Shoal-Ammen Rock 1	-2.923	0.000
Star Island-Ammen Rock 1	-3.311	0.000
Lunging Island-Ammen Rock 2	-2.706	0.000
Mingo Rock-Ammen Rock 2	-2.460	0.000
Spout Shoal-Ammen Rock 2	-3.047	0.000
Star Island-Ammen Rock 2	-3.435	0.000
Mingo Rock-Lunging Island	0.245	0.905
Spout Shoal-Lunging Island	-0.341	0.707
Star Island-Lunging Island	-0.729	0.040
Spout Shoal-Mingo Rock	-0.586	0.156
Star Island-Mingo Rock	-0.974	0.002
Star Island-Spout Shoal	-0.388	0.583

One-way ANOVA Factor: Site
Response Variable: 2015 S. digitata density per 1.0 m2 (log(x + 1.1) transformed)

ANOVA Table

	Df	Sum of Squares	Mean Square	F for Model	Pr(>F)
Site	5	1.510	0.302	1.156	0.345
Residuals	47	12.300	0.261		

One-way ANOVA Factor: Site
Response Variable: 2015 A. clathratum density per 1.0 m2 (log(x + 1.1) transformed)

ANOVA Table

	Df	Sum of Squares	Mean Square	F for Model	Pr(>F)
Site	5	115.000	22.900	688.950	<.001
Residuals	47	1.560	0.033		

Tukey Multiple Comparison of Means

Site Comparison	Mean Difference	P-value (adjusted)
Ammen Rock 2-Ammen Rock 1	0.129	0.690
Lunging Island-Ammen Rock 1	2.500	0.000
Mingo Rock-Ammen Rock 1	3.524	0.000

Spout Shoal-Ammen Rock 1	3.082	0.000
Star Island-Ammen Rock 1	2.956	0.000
Lunging Island-Ammen Rock 2	2.371	0.000
Mingo Rock-Ammen Rock 2	3.395	0.000
Spout Shoal-Ammen Rock 2	2.952	0.000
Star Island-Ammen Rock 2	2.827	0.000
Mingo Rock-Lunging Island	1.024	0.000
Spout Shoal-Lunging Island	0.582	0.000
Star Island-Lunging Island	0.456	0.003
Spout Shoal-Mingo Rock	-0.443	0.005
Star Island-Mingo Rock	-0.568	0.000
Star Island-Spout Shoal	-0.125	0.884

One-way ANOVA Factor: Site
Response Variable: 2014 S. latissima density per 1.0 m2 (log(x + 1.1) transformed)

ANOVA Table

	Df	Sum of Squares	Mean Square	F for Model	Pr(>F)
Site	5	260.000	51.900	359.750	<.001
Residuals	124	17.900	0.144		

Tukey Multiple Comparison of Means

**Site Comparison**	**Mean Difference**	**P-value (adjusted)**
Duck Island-Ammen Rock 1	-2.851	0.000
Lunging Island-Ammen Rock 1	-3.576	0.000
Mingo Rock-Ammen Rock 1	-3.373	0.000
Spout Shoal-Ammen Rock 1	-3.401	0.000
Star Island-Ammen Rock 1	-3.415	0.000
Lunging Island-Duck Island	-0.725	0.000
Mingo Rock-Duck Island	-0.522	0.001
Spout Shoal-Duck Island	-0.549	0.000
Star Island-Duck Island	-0.563	0.000
Mingo Rock-Lunging Island	0.203	0.731
Spout Shoal-Lunging Island	0.176	0.809
Star Island-Lunging Island	0.162	0.868
Spout Shoal-Mingo Rock	-0.028	1.000
Star Island-Mingo Rock	-0.042	0.999
Star Island-Spout Shoal	-0.014	1.000

One-way ANOVA Factor: Site
Response Variable: 2014 S. digitata density per 1.0 m2 (log(x + 1.1) transformed)

ANOVA Table

	Df	Sum of Squares	Mean Square	F for Model	Pr(>F)
Site	5	0.000	0.000	0.658	0.656
Residuals	124	0.000	0.000		

One-way ANOVA Factor: Site
Response Variable: 2014 A. clathratum density per 1.0 m2 (log(x + 1.1) transformed)

ANOVA Table

	Df	Sum of Squares	Mean Square	F for Model	Pr(>F)
Site	5	50.200	10.000	25.526	<.001
Residuals	124	48.800	0.394		

Tukey Multiple Comparison of Means

Site Comparison	Mean Difference	P-value (adjusted)
Duck Island-Ammen Rock 1	1.063	0.000
Lunging Island-Ammen Rock 1	2.075	0.000
Mingo Rock-Ammen Rock 1	0.122	0.983
Spout Shoal-Ammen Rock 1	0.572	0.009
Star Island-Ammen Rock 1	1.241	0.000
Lunging Island-Duck Island	1.011	0.001
Mingo Rock-Duck Island	-0.941	0.000
Spout Shoal-Duck Island	-0.491	0.119
Star Island-Duck Island	0.178	0.947
Mingo Rock-Lunging Island	-1.952	0.000
Spout Shoal-Lunging Island	-1.502	0.000
Star Island-Lunging Island	-0.833	0.008
Spout Shoal-Mingo Rock	0.450	0.137
Star Island-Mingo Rock	1.119	0.000
Star Island-Spout Shoal	0.669	0.003

Ammen Rock 1 temporal comparison

Ammen Rock 1
One-way ANOVA Factor: Year
Response Variable: S. latissima density per 1.0 m2 (log(x + 1.1) transformed)

ANOVA Table

	Df	Sum of Squares	Mean Square	F for Model	Pr(>F)
Year	3	2.310	0.770	5.734	0.001
Residuals	118	15.800	0.134		

Tukey Multiple Comparison of Means

Site Comparison	Mean Difference	P-value (adjusted)
2012-1987	-0.243	0.055
2014-1987	-0.138	0.515
2015-1987	-0.404	0.001
2014-2012	0.105	0.639
2015-2012	-0.161	0.286
2015-2014	-0.266	0.033

One-way ANOVA Factor: Year
Response Variable: S. digitata density per 1.0 m2 (log(x + 1.1) transformed)

ANOVA Table

	Df	Sum of Squares	Mean Square	F for Model	Pr(>F)
Year	3	2.620	0.873	9.272	<.001
Residuals	118	11.100	0.094		

Tukey Multiple Comparison of Means

Site Comparison	Mean Difference	P-value (adjusted)
2012-1987	0.000	1.000
2014-1987	0.000	1.000
2015-1987	0.348	0.000
2014-2012	0.000	1.000
2015-2012	0.348	0.000
2015-2014	0.348	0.000

One-way ANOVA Factor: Year
Response Variable: A. clathratum density per 1.0 m2 (log(x + 1.1) transformed)

ANOVA Table

	Df	Sum of Squares	Mean Square	F for Model	Pr(>F)
Year	3	0.000	0.000	1.122	0.343
Residuals	118	0.000	0.000		
