# Supplementary material for: Persistent differences between coastal and offshore kelp forest communities in a warming Gulf of Maine
Source: PLoS One. 2018 Jan 3;13(1):e0189388. doi: 10.1371/journal.pone.0189388 (PMC5751975; doi:10.1371/journal.pone.0189388)
Supplement: S5 Table — Data are means with standard deviations (SD). (PDF) [file pone.0189388.s008.pdf]

S5 Table Percent cover of *Membranipora membranacea* on kelp at Cashes Ledge. Data are means with standard deviations (SD).

**Site**	**Year**	***Membranipora* cover**	**Sample Size (n)**
Ammen Rock 1	1987	0	12
Ammen Rock 1	2012	27.9 (8.5)	11
Ammen Rock 1	2014	0.3 (0.5)	22
Ammen Rock 1	2015	5.7(10.2)	29
Ammen Rock 2	2015	0.04 (0.006)	23
