# Supplementary material for: Persistent differences between coastal and offshore kelp forest communities in a warming Gulf of Maine
Source: PLoS One. 2018 Jan 3;13(1):e0189388. doi: 10.1371/journal.pone.0189388 (PMC5751975; doi:10.1371/journal.pone.0189388)
Supplement: S7 Table — Data are means with Standard Errors (SE). Sample sizes are numbers of 1.0 m 2 quadrats searched. No sea urchins were found at the Ammen Rock sites 1 in 1987, 2012 and at Ammen Rock sites 2 and 3 in 2016 (n = 102, 1.0 m 2 total quadrats sampled at these sites). (PDF) [file pone.0189388.s010.pdf]

S7 Table. Density of sea urchins *S. droebachiensis* per 1.0 m^2 in 2014 and 2015. Data are means with Standard Errors (SE). Sample sizes are numbers of 1.0 m ^{2} quadrats searched. No sea urchins were found at the Ammen Rock sites 1 in 1987, 2012 and at Ammen Rock sites 2 and 3 in 2016 (n = 102, 1.0 m ^{2} total quadrats sampled at these sites).

**2014**		
**Site**	**Urchin Density**	**Sample Size (n)**
Duck Island (DI)	1.3 (0.57)	10
Mingo Rock (MR)	0.8 (0.29)	11
Spout Shoal (SS)	1.0 (0.39)	10
Star Island (SI)	3.5 (1.3)	10
Ammen Rock 1 (AR1)	0	10
**2015**		
Mingo Rock (MR)	0.6 (0.22)	10

Spout Shoal (SS)	1.0 (0.42)	10
Star Island (SI)	36.2 (2.0)	10
Ammen Rock 1 (AR1)	0.5 (0.5)	20
Ammen Rock 2 (AR2)	0	14
