# Supplementary material for: Persistent differences between coastal and offshore kelp forest communities in a warming Gulf of Maine
Source: PLoS One. 2018 Jan 3;13(1):e0189388. doi: 10.1371/journal.pone.0189388 (PMC5751975; doi:10.1371/journal.pone.0189388)
Supplement: S12 Table — Data used are biomass (g/50m2). We performed a two-way ANOVA to test for significant differences between sites and years and their interaction, followed by Tukey’s Honest Significant Difference test for pairwise comparisons. (PDF) [file pone.0189388.s015.pdf]

S12 Table. Temporal and spatial comparison of total fish biomass, followed by post-hoc tests. Data used are biomass (g/50m^2). We performed a two-way ANOVA to test for significant differences between sites and years and their interaction, followed by Tukey's Honest Significant Difference test for pairwise comparisons.

S12 Table. (A). ANOVA comparing total fish biomass of fish by Year (2014 - 2015) and Site

	Df	Sum of Squares	Mean Square	F for Model	Pr(>F)
Site	6	1158.410	193.068	106.999	<.001
Year	1	7.280	7.277	4.033	0.048
Site:Year	3	26.950	8.982	4.978	0.003
Residuals	83	149.760	1.804		

S12 Table. (B). Post hoc comparison of means by Tukey's HSD test.

Comparison	Difference	Lower bound	Upper bound	Adjusted p-value
Ammen Rock 2-Ammen Rock 1	-0.710	-2.410	0.990	0.868
Duck Island-Ammen Rock 1	-8.359	-10.402	-6.316	<.001
Lunging Island-Ammen Rock 1	-6.374	-8.074	-4.674	<.001
Mingo Rock-Ammen Rock 1	-8.227	-9.653	-6.800	<.001
Spout Shoal-Ammen Rock 1	-8.113	-9.677	-6.549	<.001
Star Island-Ammen Rock 1	-7.198	-8.646	-5.750	<.001
Duck Island-Ammen Rock 2	-7.649	-9.916	-5.382	<.001
Lunging Island-Ammen Rock 2	-5.664	-7.627	-3.701	<.001
Mingo Rock-Ammen Rock 2	-7.516	-9.248	-5.785	<.001
Spout Shoal-Ammen Rock 2	-7.403	-9.249	-5.556	<.001
Star Island-Ammen Rock 2	-6.488	-8.238	-4.739	<.001
Lunging Island-Duck Island	1.985	-0.282	4.251	0.126
Mingo Rock-Duck Island	0.132	-1.937	2.202	1.000

Spout Shoal-Duck Island	0.246	-1.920	2.413	1.000
Star Island-Duck Island	1.161	-0.924	3.245	0.631
Mingo Rock-Lunging Island	-1.852	-3.584	-0.121	0.028
Spout Shoal-Lunging Island	-1.739	-3.585	0.108	0.079
Star Island-Lunging Island	-0.824	-2.573	0.925	0.789
Spout Shoal-Mingo Rock	0.114	-1.484	1.711	1.000
Star Island-Mingo Rock	1.028	-0.456	2.513	0.367
Star Island-Spout Shoal	0.914	-0.703	2.532	0.613

S12 Table. (C). Tukey's HSD post-hoc test (Site*Year)

**Comparison**	**Difference**	**Lower bound**	**Upper bound**	**Adjusted p-value**
Mingo Rock:2014-Ammen Rock 1:2014	-7.837	-9.676	-5.997	<.001
Spout Shoal:2014-Ammen Rock 1:2014	-8.641	-11.194	-6.088	<.001
Star Island:2014-Ammen Rock 1:2014	-5.492	-7.403	-3.581	<.001
Ammen Rock 1:2015-Ammen Rock 1:2014	1.730	-0.004	3.465	0.051
Mingo Rock:2015-Ammen Rock 1:2014	-6.981	-8.715	-5.247	<.001
Spout Shoal:2015-Ammen Rock 1:2014	-6.829	-8.564	-5.095	<.001
Star Island:2015-Ammen Rock 1:2014	-6.922	-8.656	-5.188	<.001
Spout Shoal:2014-Mingo Rock:2014	-0.805	-3.430	1.821	0.978
Star Island:2014-Mingo Rock:2014	2.345	0.338	4.352	0.011
Ammen Rock 1:2015-Mingo Rock:2014	9.567	7.728	11.406	<.001
Mingo Rock:2015-Mingo Rock:2014	0.855	-0.984	2.695	0.825
Spout Shoal:2015-Mingo Rock:2014	1.007	-0.832	2.847	0.675
Star Island:2015-Mingo Rock:2014	0.915	-0.925	2.754	0.771
Star Island:2014-Spout Shoal:2014	3.149	0.473	5.826	0.011

Ammen Rock 1:2015-Spout Shoal:2014	10.372	7.819	12.925	<.001
Mingo Rock:2015-Spout Shoal:2014	1.660	-0.893	4.213	0.463
Spout Shoal:2015-Spout Shoal:2014	1.812	-0.741	4.365	0.350
Star Island:2015-Spout Shoal:2014	1.719	-0.834	4.272	0.417
Ammen Rock 1:2015-Star Island:2014	7.222	5.311	9.133	<.001
Mingo Rock:2015-Star Island:2014	-1.489	-3.400	0.422	0.238
Spout Shoal:2015-Star Island:2014	-1.338	-3.249	0.573	0.368
Star Island:2015-Star Island:2014	-1.430	-3.341	0.481	0.285
Mingo Rock:2015-Ammen Rock 1:2015	-8.712	-10.446	-6.977	<.001
Spout Shoal:2015-Ammen Rock 1:2015	-8.560	-10.294	-6.826	<.001
Star Island:2015-Ammen Rock 1:2015	-8.652	-10.387	-6.918	<.001
Spout Shoal:2015-Mingo Rock:2015	0.152	-1.583	1.886	1.000
Star Island:2015-Mingo Rock:2015	0.059	-1.675	1.794	1.000
Star Island:2015-Spout Shoal:2015	-0.093	-1.827	1.642	1.000
