# Supplementary material for: Persistent differences between coastal and offshore kelp forest communities in a warming Gulf of Maine
Source: PLoS One. 2018 Jan 3;13(1):e0189388. doi: 10.1371/journal.pone.0189388 (PMC5751975; doi:10.1371/journal.pone.0189388)
Supplement: S13 Table — Data used are MaxN: maximum number of individuals per species observed in a 10-minute segment of stationary video. For each species, we performed an ANOVA test for significant differences between sites, followed by Tukey’s Honest Significant Difference test for pairwise comparisons. (PDF) [file pone.0189388.s016.pdf]

**S13 Table. Spatial comparison of fish abundance (cod, cunner, and pollock) by site, followed by post-hoc tests.** Data used are MaxN: maximum number of individuals per species observed in a 10-minute segment of stationary video. For each species, we performed an ANOVA test for significant differences between sites, followed by Tukey's Honest Significant Difference test for pairwise comparisons.

S13 Table. (A) ANOVA test of effect of site on cod abundance (MaxN)

| | Df | Sum of Squares | Mean Square | F for Model | Pr(>F) |
|---|---|---|---|---|---|
| Site | 6 | 42.064 | 7.0107 | 64.637 | <.001 |
| Residuals | 106 | 11.497 | 0.1085 | | |

S13 Table. (B). Tukey's HSD test for pairwise differences in cod abundance (all years combined)

| Comparison | Difference | Lower bound | Upper bound | Adjusted p-value |
|---|---|---|---|---|
| Ammen Rock 2-Ammen Rock 1 | 0.688 | 0.394 | 0.982 | <.001 |
| Ammen Rock 3-Ammen Rock 1 | 0.542 | 0.094 | 0.991 | 0.008 |
| Lunging Island-Ammen Rock 1 | -0.822 | -1.159 | -0.486 | <.001 |
| Mingo Rock-Ammen Rock 1 | -0.822 | -1.131 | -0.514 | <.001 |
| Spout Shoal-Ammen Rock 1 | -0.769 | -1.105 | -0.433 | <.001 |
| Star Island-Ammen Rock 1 | -0.822 | -1.126 | -0.519 | <.001 |
| Ammen Rock 3-Ammen Rock 2 | -0.146 | -0.606 | 0.315 | 0.963 |
| Lunging Island-Ammen Rock 2 | -1.51 | -1.863 | -1.158 | <.001 |
| Mingo Rock-Ammen Rock 2 | -1.51 | -1.837 | -1.184 | <.001 |
| Spout Shoal-Ammen Rock 2 | -1.457 | -1.81 | -1.104 | <.001 |
| Star Island-Ammen Rock 2 | -1.51 | -1.832 | -1.189 | <.001 |
| Lunging Island-Ammen Rock 3 | -1.365 | -1.853 | -0.876 | <.001 |
| Mingo Rock-Ammen | -1.365 | -1.835 | -0.895 | <.001 |

| | | | | |
|---|---|---|---|---|
| Rock 3 | | | | |
| Spout Shoal-Ammen Rock 3 | -1.311 | -1.8 | -0.823 | <.001 |
| Star Island-Ammen Rock 3 | -1.365 | -1.831 | -0.898 | <.001 |
| Mingo Rock-Lunging Island | 0 | -0.365 | 0.365 | 1 |
| Spout Shoal-Lunging Island | 0.053 | -0.335 | 0.442 | 1 |
| Star Island-Lunging Island | 0 | -0.36 | 0.36 | 1 |
| Spout Shoal-Mingo Rock | 0.053 | -0.311 | 0.418 | 0.999 |
| Star Island-Mingo Rock | 0 | -0.335 | 0.335 | 1 |
| Star Island-Spout Shoal | -0.053 | -0.414 | 0.307 | 0.999 |

S13 Table. (C) ANOVA test of effect of site on cunner abundance (MaxN)

| | Df | Sum of Squares | Mean Square | F for Model | Pr(>F) |
|---|---|---|---|---|---|
| Site | 6 | 168.095 | 28.0158 | 95.402 | <.001 |
| Residuals | 106 | 31.128 | 0.2937 | | |

S13 Table. (D) Tukey's honest significant test for pairwise differences in cunner abundance (all years combined)

| Comparison | Difference | Lower bound | Upper bound | Adjusted p-value |
|---|---|---|---|---|
| Ammen Rock 2-Ammen Rock 1 | 1.13 | 0.645 | 1.614 | <.001 |
| Ammen Rock 3-Ammen Rock 1 | 1.558 | 0.82 | 2.295 | <.001 |
| Lunging Island-Ammen Rock 1 | -1.026 | -1.579 | -0.472 | <.001 |
| Mingo Rock-Ammen Rock 1 | -2.119 | -2.627 | -1.611 | <.001 |
| Spout Shoal-Ammen Rock 1 | -1.662 | -2.215 | -1.108 | <.001 |
| Star Island-Ammen Rock 1 | -1.505 | -2.005 | -1.006 | <.001 |
| Ammen Rock 3-Ammen Rock 2 | 0.428 | -0.33 | 1.186 | 0.619 |
| Lunging Island-Ammen Rock 2 | -2.155 | -2.736 | -1.575 | <.001 |

| | | | | |
|---|---|---|---|---|
| Mingo Rock-Ammen Rock 2 | -3.249 | -3.786 | -2.711 | <.001 |
| Spout Shoal-Ammen Rock 2 | -2.791 | -3.371 | -2.211 | <.001 |
| Star Island-Ammen Rock 2 | -2.635 | -3.164 | -2.106 | <.001 |
| Lunging Island-Ammen Rock 3 | -2.583 | -3.387 | -1.78 | <.001 |
| Mingo Rock-Ammen Rock 3 | -3.677 | -4.45 | -2.904 | <.001 |
| Spout Shoal-Ammen Rock 3 | -3.219 | -4.023 | -2.415 | <.001 |
| Star Island-Ammen Rock 3 | -3.063 | -3.831 | -2.295 | <.001 |
| Mingo Rock-Lunging Island | -1.094 | -1.694 | -0.493 | <.001 |
| Spout Shoal-Lunging Island | -0.636 | -1.275 | 0.003 | 0.052 |
| Star Island-Lunging Island | -0.48 | -1.072 | 0.113 | 0.196 |
| Spout Shoal-Mingo Rock | 0.458 | -0.142 | 1.058 | 0.257 |
| Star Island-Mingo Rock | 0.614 | 0.063 | 1.165 | 0.019 |
| Star Island-Spout Shoal | 0.156 | -0.437 | 0.749 | 0.985 |

S13 Table. (E) ANOVA test of effect of site on pollock abundance (MaxN)

| | **Df** | **Sum of Squares** | **Mean Square** | **F for Model** | **Pr(>F)** |
|---|---|---|---|---|---|
| Site | 6 | 40.714 | 6.786 | 8.061 | <.001 |
| Residuals | 106 | 89.234 | 0.842 | | |

S13 Table. (F) Tukey's honest significant test for pairwise differences in cunner abundance (all years combined)

| **Comparison** | **Difference** | **Lower bound** | **Upper bound** | **Adjusted p-value** |
|---|---|---|---|---|
| Ammen Rock 2-Ammen Rock 1 | 0.332 | -0.488 | 1.153 | 0.886 |
| Ammen Rock 3-Ammen Rock 1 | 0.134 | -1.115 | 1.383 | 1 |
| Lunging Island-Ammen Rock 1 | -0.686 | -1.623 | 0.251 | 0.304 |

| | | | | |
|---|---|---|---|---|
| Mingo Rock-Ammen Rock 1 | 0.109 | -0.751 | 0.969 | 1 |
| Spout Shoal-Ammen Rock 1 | 1.008 | 0.071 | 1.944 | 0.026 |
| Star Island-Ammen Rock 1 | -1.03 | -1.876 | -0.185 | 0.007 |
| Ammen Rock 3-Ammen Rock 2 | -0.199 | -1.482 | 1.085 | 0.999 |
| Lunging Island-Ammen Rock 2 | -1.018 | -2.001 | -0.036 | 0.037 |
| Mingo Rock-Ammen Rock 2 | -0.223 | -1.133 | 0.686 | 0.99 |
| Spout Shoal-Ammen Rock 2 | 0.675 | -0.307 | 1.658 | 0.381 |
| Star Island-Ammen Rock 2 | -1.363 | -2.259 | -0.467 | <.001 |
| Lunging Island-Ammen Rock 3 | -0.82 | -2.181 | 0.541 | 0.544 |
| Mingo Rock-Ammen Rock 3 | -0.025 | -1.334 | 1.285 | 1 |
| Spout Shoal-Ammen Rock 3 | 0.874 | -0.487 | 2.235 | 0.466 |
| Star Island-Ammen Rock 3 | -1.164 | -2.464 | 0.136 | 0.111 |
| Mingo Rock-Lunging Island | 0.795 | -0.221 | 1.811 | 0.23 |
| Spout Shoal-Lunging Island | 1.693 | 0.612 | 2.775 | <.001 |
| Star Island-Lunging Island | -0.344 | -1.348 | 0.659 | 0.946 |
| Spout Shoal-Mingo Rock | 0.899 | -0.117 | 1.915 | 0.12 |
| Star Island-Mingo Rock | -1.139 | -2.072 | -0.207 | 0.007 |
| Star Island-Spout Shoal | -2.038 | -3.042 | -1.034 | <.001 |
