# Supplementary material for: Persistent differences between coastal and offshore kelp forest communities in a warming Gulf of Maine
Source: PLoS One. 2018 Jan 3;13(1):e0189388. doi: 10.1371/journal.pone.0189388 (PMC5751975; doi:10.1371/journal.pone.0189388)
Supplement: S14 Table — Data used are MaxN: maximum number of individuals per species observed in a 10-minute segment of stationary video. For each species, we performed an ANOVA test for significant differences between years, followed by Tukey’s Honest Significant Difference test for pairwise comparisons. (PDF) [file pone.0189388.s017.pdf]

**S14 Table. Temporal comparison of fish abundance (cod, cunner, and pollock) by year (2014, 2015, and 2016) at Cashes Ledge sites, followed by post-hoc tests.** Data used are MaxN: maximum number of individuals per species observed in a 10-minute segment of stationary video. For each species, we performed an ANOVA test for significant differences between years, followed by Tukey's Honest Significant Difference test for pairwise comparisons.

ANOVA test of effect of year on cod abundance at Cashes Ledge (MaxN)

| | **Df** | **Sum of squares** | **Mean square** | **F for Model** | **Pr(>F)** |
|---|---|---|---|---|---|
| Year | 2 | 0.569 | 0.285 | 0.903 | 0.413 |
| Residuals | 45 | 14.183 | 0.315 | | |

Tukey's HSD test for pairwise differences between years in cod abundance at Cashes Ledge

| **Comparison** | **Difference** | **Lower bound** | **Upper bound** | **Adjusted p-value** |
|---|---|---|---|---|
| 2015-2014 | 0.315 | -0.267 | 0.897 | 0.396 |
| 2016-2014 | 0.296 | -0.32 | 0.913 | 0.48 |
| 2016-2015 | -0.019 | -0.454 | 0.417 | 0.994 |

ANOVA test of effect of year on cunner abundance at Cashes Ledge (MaxN)

| | **Df** | **Sum of squares** | **Mean square** | **F for Model** | **Pr(>F)** |
|---|---|---|---|---|---|
| Year | 2 | 14.39 | 7.1952 | 26.08 | <.001 |
| Residuals | 45 | 12.415 | 0.2759 | | |

Tukey's honest significant test for pairwise differences between years in cunner abundance at Cashes Ledge

| **Comparison** | **Diffference** | **Lower bound** | **Upper bound** | **Adjusted p-value** |
|---|---|---|---|---|
| 2015-2014 | 0.479 | -0.065 | 1.023 | 0.094 |
| 2016-2014 | 1.484 | 0.907 | 2.061 | <.001 |
| 2016-2015 | 1.005 | 0.597 | 1.412 | <.001 |

ANOVA test of effect of year on pollack abundance at Cashes Ledge (MaxN)

| | **Df** | **Sum of squares** | **Mean square** | **F for Model** | **Pr(>F)** |
|---|---|---|---|---|---|
| Year | 2 | 4.316 | 2.158 | 4.667 | 0.014 |
| Residuals | 45 | 20.811 | 0.462 | | |

Tukey's honest significant test for pairwise differences between years in pollack abundance at Cashes Ledge

| Comparison | Diffference | Lower bound | Upper bound | Adjusted p-value |
|---|---|---|---|---|
| 2015-2014 | -0.814 | -1.519 | -0.109 | 0.02 |
| 2016-2014 | -0.891 | -1.638 | -0.144 | 0.016 |
| 2016-2015 | -0.076 | -0.604 | 0.451 | 0.934 |
