# Supplementary material for: Persistent differences between coastal and offshore kelp forest communities in a warming Gulf of Maine
Source: PLoS One. 2018 Jan 3;13(1):e0189388. doi: 10.1371/journal.pone.0189388 (PMC5751975; doi:10.1371/journal.pone.0189388)
Supplement: S15 Table — We compared kelp density and biomass on Cashes Ledge to kelp abundance within the same depth range (12–15 m) and kelp abundance at shallower depths (6–10 m) at other sites in the NW Atlantic. Multiple entries from the same site are from different sampling dates. Saccharina longicruris = S. latissima; Laminaria digitata = S. digitata. Data are from literature review: Krumhansl et al. 2016, and this study. (PDF) [file pone.0189388.s018.pdf]

S 15 Table. Comparison of kelp abundance (density, biomass) on Cashes Ledge to A. kelp abundance within the same depth range (12 to 15 , this study) and to kelp abundance at shallower depths (6-10 m) at other sites in the NW Atlantic. Multiple entries for the same site are from different sampling dates. *Saccharina longicruris = S. latissima*
Data are from literature review, Krumhansl et al. 2016*, and this study.

A. Kelp in same depth range as this study (12 to 15 m)

Reference	Species	nsity (indivuals / 1.0 m	Biomass (kg/ 1.0 m2)	Depth (m)	Study Site and Year Sampled
Witman 1984	*Saccharina digitata*	15.60		12.5	Aurray Rock, Gulf of Maine , 1981
	Saccharina latissima	8.00		12.5	Aurray Rock, Gulf of Maine , 1981
	Agarum clathratum	9.00		12.5	Aurray Rock, Gulf of Maine , 1981
Bologna and Steneck 1993*	*L. saccharina and L. longicruris*	12.70		10 to 15	Crow Island, Gulf of Maine , 1990
	L. saccharina and L. longicruris	40.60		10 to 15	Crow Island, Gulf of Maine , 1990
Saunders and Metaxas 2009	*Saccharina latissima*	2.67		12	Lodge, Nova Scotia, 2005
	Laminaria digitata	0.00		12	Lodge, Nova Scotia, 2005
	Agarum clathratum	2.56		12	Lodge, Nova Scotia, 2005
	Saccharina latissima	0.15		12	Lodge, Nova Scotia, 2007
	Laminaria digitata	0.00		12	Lodge, Nova Scotia, 2007
	Agarum clathratum	0.80		12	Lodge, Nova Scotia, 2007
	Saccharina latissima	1.73		12	Paddy's Head, Nova Scotia, 2005
	Laminaria digitata	0.45		12	Paddy's Head, Nova Scotia, 2005
	Agarum clathratum	6.73		12	Paddy's Head, Nova Scotia, 2005
	Saccharina latissima	0.50		12	Paddy's Head, Nova Scotia, 2007
	Laminaria digitata	0.05		12	Paddy's Head, Nova Scotia, 2007
	Agarum clathratum	2.85		12	Paddy's Head, Nova Scotia, 2007
	Saccharina latissima	2.00		12	Sandy Cove, Nova Scotia, 2005
	Laminaria digitata	0.70		12	Sandy Cove, Nova Scotia, 2005
	Agarum clathratum	3.15		12	Sandy Cove, Nova Scotia, 2005
Krumhansl and Scheibling 2011	*Saccharina latissima*	12.4	0.33	11	Lodge, Nova Scotia, 2008
	Laminaria digitata	2.4	0.16	11	Lodge, Nova Scotia, 2008
Witman and Lamb, this study	*Saccharina latissima*	47.80		12 to 15	ıes Ledge, Ammen Rock Site 1, 1987
	Saccharina latissima	36.82	2.38	12 to 15	ıes Ledge, Ammen Rock Site 1, 2012
	Saccharina latissima	40.56	5.55	12 to 15	ıes Ledge, Ammen Rock Site 1, 2014
	Saccharina latissima	30.40	4.82	12 to 15	ıes Ledge, Ammen Rock Site 1, 2015
	Saccharina digitata	0.96	0.22	12 to 15	ıes Ledge, Ammen Rock Site 1, 2015
	Saccharina latissima	33.60	3.43	12 to 15	ıes Ledge, Ammen Rock Site 2, 2015
	Agarum clathratum	0.20	0.11	12 to 15	ıes Ledge, Ammen Rock Site 2, 2015
	Saccharina latissima	10.50	2.08	12 to 15	ıes Ledge, Ammen Rock Site 2, 2016
	Saccharina digitata	26.20	1.85	12 to 15	ıes Ledge, Ammen Rock Site 2, 2016
	Saccharina latissima	13.50		12 to 15	ıes Ledge, Ammen Rock Site 3 , 2016
	Saccharina digitata	2.50		12 to 15	ıes Ledge, Ammen Rock Site 3, 2016

B. Kelp at shallower depths (6 - 10 m) than this study

Reference	Species	nsity (indivuals / 1.0 m	Biomass (kg/ 1.0 m2)	Depth (m)	Study Site and Year Sampled
Scheibling 1986	*Saccharina latissima*	1.00	1.00	9	Vhitehead Island, Nova Scotia, 1984
	Laminaria digitata	2.75	2.75	9	Vhitehead Island, Nova Scotia, 1984
Lauzon-Guay and Scheibling 2007	*Saccharina latissima*	9.90	3.5	10	plitnose Point, Nova Scotia, 2005
	Laminaria digitata	8.10	3.1	10	plitnose Point, Nova Scotia, 2005
Krumhansl and Scheibling 2011	*Saccharina latissima*	7.37	0.48	6	Lodge, Nova Scotia, 2008
	Saccharina latissima	6.5	0.46	6	Lodge, Nova Scotia, 2008
	Saccharina latissima	12.4	0.33	6	Lodge, Nova Scotia, 2008
	Saccharina latissima	16	2.28	6	Lodge, Nova Scotia, 2009
	Saccharina latissima	15.11	1.83	6	Lodge, Nova Scotia, 2009
	Laminaria digitata	4.32	0.49	6	Lodge, Nova Scotia, 2009
	Laminaria digitata	1	0.14	6	Lodge, Nova Scotia, 2009
	Laminaria digitata	2.4	0.16	6	Lodge, Nova Scotia, 2009
	Laminaria digitata	1.56	0.05	6	Lodge, Nova Scotia, 2009
	Laminaria digitata	2.22	0.36	6	Lodge, Nova Scotia, 2009
Scheibling and Gagnon 2009 , Kelly et al. 2011	*Saccharina latissima*	38	0.5	6	Little Duck Island, Nova Scotia, 1992
	Saccharina latissima	21	3.2	6	Little Duck Island, Nova Scotia, 1993
	Saccharina latissima	40	0.9	6	Little Duck Island, Nova Scotia, 1994
	Saccharina latissima	45	5.8	6	Little Duck Island, Nova Scotia, 1995
	Saccharina latissima	26	2.8	6	Little Duck Island, Nova Scotia, 1996
	Saccharina latissima	60	2.2	6	Little Duck Island, Nova Scotia, 1997
Krumhansl and Scheibling 2011	*Saccharina latissima*	42.89	2.18	6	Cranberry Cove, Nova Scotia , 2008
	Saccharina latissima	37.11	1.66	6	Cranberry Cove, Nova Scotia , 2008
	Saccharina latissima	51.56	1.32	6	Cranberry Cove, Nova Scotia , 2009
	Saccharina latissima	55.56	2.17	6	Cranberry Cove, Nova Scotia , 2009
	Saccharina latissima	37.78	1.52	6	Cranberry Cove, Nova Scotia , 2009
	Laminaria digitata	1.56	0.64	6	Cranberry Cove, Nova Scotia , 2009
	Laminaria digitata	0.11	0.1	6	Cranberry Cove, Nova Scotia , 2009
	Laminaria digitata	8.78	0.25	6	Cranberry Cove, Nova Scotia , 2009
	Laminaria digitata	1.33	0.02	6	Cranberry Cove, Nova Scotia , 2009
	Laminaria digitata	1.56	0.04	6	Cranberry Cove, Nova Scotia , 2009

* Krumhansl, K. A., D. K. Okamoto, A. Rassweiler, M. Novak, J. J. Bolton, K. C. Cavanaugh, S. D. Connell, C. R. Johnson, B. Konar, S. D. Ling et al. 2016
Global patterns of kelp forest change over the past half century. Proceedings of the National Academy of Sciences. www.pnas.org/cgi/doi/10.1073/pnas.1606102113

* Bologna and Steneck 1993 combined data for both species
