# Supplementary material for: Persistent differences between coastal and offshore kelp forest communities in a warming Gulf of Maine
Source: PLoS One. 2018 Jan 3;13(1):e0189388. doi: 10.1371/journal.pone.0189388 (PMC5751975; doi:10.1371/journal.pone.0189388)
Supplement: S16 Table — This analysis was performed on our UVC data standardized to 500m2 for comparison with other sampling efforts in the region (Stuart-Smith et al., 2013). Fish density at Cashes Ledge averaged 1227.5 (246.6 SE) individuals per 500m2, compared to only 24.8 (7.1 SE) individuals at coastal sites. Densities at Cashes Ledge were more than twice the average reported for the entire North Atlantic, whereas values at our coastal sites were less that 5% of the regional average (Stuart-Smith et al. 2013). (PDF) [file pone.0189388.s019.pdf]

S 16 Table This analysis was performed on our UVC data standardized to 500m^2 for comparison with other sampling efforts in the region (Stuart-Smith et al., 2013). Fish density at Cashes Ledge averaged 1227.5 (246.6 SE) individuals per 500m^2, compared to only 24.8 (7.1 SE) individuals at coastal sites. Densities at Cashes Ledge were more than twice the average reported for the entire North Atlantic, whereas values at our coastal sites were less that 5% of the regional average (Stuart-Smith et al. 2013*).

Site	Mean total fish density (500 m^2)	SE
Ammen Rock 1	1038.9	287.7
Ammen Rock 2	1699.0	371.0
**Cashes Ledge**	**1227.5**	**246.6**
Duck Island	8.3	1.7
Lunging Island	78.0	32.0
Mingo Rock	10.5	4.0
Spout Shoal	10.0	5.8
Star Island	30.5	9.4
**Coastal**	**24.8**	**7.1**

*Stuart Smith RD, Bates AE, Lefcheck JS, Duffy JE, Baker SC, Thomson RJ, et al. Integrating abundance and functional traits reveals new global hotspots of fish diversity. 2014. Nature 501, 539-542
